# Global trends in fall-induced hip fractures among perimenopausal women 1990 to 2021: results from the global burden of disease study 2021

**DOI:** 10.3389/fpubh.2025.1674535

**Published:** 2025-10-14

**Authors:** Zhen Wang, Zijian Chen, Chaoyi Zhang, Wenzheng Liu, Jixi Liu, Wei Lin, Guanglin Wang

**Affiliations:** ^1^Department of Orthopedics Surgery, West China Hospital, Trauma Medical Center, Sichuan University, Chengdu, China; ^2^Department of Orthopedics, West China Hospital, Sichuan University, Chengdu, China; ^3^Department of Gynecology, West China Second Hospital, Sichuan University, Chengdu, China

**Keywords:** perimenopausal women, falls, hip fractures, orthopedics, global burden of disease

## Abstract

**Background:**

Fall-induced hip fractures in perimenopausal women (FHFPW) are major clinical and public health concern, contributing significantly to global morbidity. This study examines the global impact of FHFPW, focusing on the prevalence, incidence, and years lived with disability (YLDs) across different age groups, regions, and levels of Socio-demographic index (SDI) from 1990 to 2021. The aim is to provide epidemiological data that can inform health policies, and enhance strategies for the prevention and treatment of hip fractures in perimenopausal women.

**Methods:**

We extracted registered data on incidence cases, prevalence cases, and YLD cases, along with the corresponding rates and their 95% uncertainty intervals (UIs), from the Global Burden of Disease (GBD) 2021 database. All rates in this study were expressed per 100,000 population. The estimated annual percentage change (EAPC) was used to assess changes in the burden of FHFPW and Spearman’s correlation tests were used to examine the relationship between SDI and the burden of FHFPW. The average annual percentage change (AAPC) was further calculated to evaluate temporal trends.

**Results:**

The disease burden of FHFPW was substantially higher in the 50-54-year age group than in the 45-49-year group. In 2021, there were 300,104 cases among women aged 50–54 years, compared with 164,196 cases in the 45-49-year group. From 1990 to 2021, although the absolute numbers of prevalent cases, incident cases, and YLDs increased, the corresponding incidence, prevalence, and YLDs rates declined. The burden was greater in high-SDI regions, particularly in Oceania, where the prevalence in the 50-54-year group demonstrated the most striking increase (+295.7%). Joinpoint analysis further indicated that, overall, prevalence and YLDs rates showed a downward trend, whereas incidence in the 50-54-year group within high-SDI regions exhibited the most pronounced rise (AAPC = 0.8%; *p* < 0.001).

**Conclusion:**

The global burden of disease for FHFPW remains high and tends to increase further; age is an important influencing factor. The burden of disease shows significant regional variations, with a higher burden of disease in regions with high SDI.

## 1 Introduction

Fall-induced hip fractures among perimenopausal women (FHFPW) are defined as fractures directly resulting from falls during the perimenopausal transition, a stage characterized by reduced secretion of oestrogen, dehydroepiandrosterone (DHEAS), and thyroid-stimulating hormone (TSH) (1–4). These hormonal changes exert substantial effects on bone health, increasing susceptibility to osteoporosis and fragility fractures (1–4). One study also identified lower serum levels of 25-hydroxyvitamin D as a significant risk factor for hip fractures, with an adjusted relative risk of 1.58 (5).

The clinical implications of hormonal changes extend beyond biological effects, encompassing considerable economic consequences, as exemplified by FHFPW (6, 7). A simulation of life expectancy, fracture risk, and economic impact estimated that Chinese women aged 50 years would experience an average of 0.135 hip fractures per person over their remaining lifetime. The residual lifetime risk of a hip fracture for a 50-year-old woman was estimated at 37.36%. These fractures lead to excess losses of 0.11 quality-adjusted life years (QALYs) and lifetime costs of $714.6, culminating in a total net monetary benefit loss of $1,104.4 (8). Globally, the direct annual cost of treating osteoporotic fractures in Canada, Europe, and the United States ranges from $500 billion to $6,500 billion, excluding indirect costs like disability and lost productivity (9).

The incidence of hip fractures has risen globally over the past three decades, particularly among individuals aged 55 years and older, highlighting the persistent burden of these injuries. In 2019, falls accounted for the highest proportion of grade 3 injuries leading to hip fractures in this age group (10, 11). Although studies report a decline in age-standardized hip fracture incidence in women [Annual Average Percentage Change (AAPC) = −1.1%] and a slight increase in men (AAPC = 0.1%) between 1990 and 2019. The socioeconomic and familial burden of FHFPW may be particularly pronounced. As many perimenopausal women remain active in the workforce and shoulder family responsibilities, hip fractures in this group impose not only substantial medical and rehabilitation costs but also indirect losses due to reduced productivity, increased dependency, and psychological stress (12). However, current studies have primarily focused on older populations or the overall burden of osteoporotic fractures, while the specific global burden of fall-induced hip fractures among FHFPW has not yet been systematically investigated.

This study aims to assess trends in the prevalence, incidence, and years lived with disability (YLDs) of FHFPW at global, regional, and national levels from 1990 to 2021 by using registered data in GBD 2021. These findings will provide a basis for developing targeted prevention and treatment strategies.

## 2 Methods

### 2.1 Data acquisition

The Global Burden of Disease (GBD) 2021 study utilized the latest epidemiological data and standardized methodologies to comprehensively assess health losses caused by 369 diseases, injuries, and impairments across 204 countries and territories, accounting for 88 risk factors. The study design and methods have been thoroughly detailed in previous GBD publications (13). All data in our study were obtained from the GBD 2021 registry and further analyzed.

### 2.2 Study definitions

#### 2.2.1 Disease definition

Falls are clearly defined as injury events resulting from an individual inadvertently coming to rest on the ground or another lower level, coded in International Classification of Diseases (ICD)-10 as W00-W19, with sequelae including hip fracture, traumatic brain injury, spinal cord injury, and other residual health outcomes. Hip fractures are coded as ICD-10 S72.0–72.2, and when these two codes occur within the same healthcare episode, cases of fall-related hip fracture can be identified (14, 15). It is important to note, however, that the GBD framework does not rely on strict parallel occurrence of these codes in individual records. Instead, it integrates multiple sources of information (hospital discharge data, vital registration, surveys, and other datasets) and applies modeling techniques to estimate incidence and prevalence.

#### 2.2.2 Bayesian meta-regression tool version 2.1

GBD estimates inherently carry uncertainty arising from several factors, including sampling error of input data, adjustments and standardization methods, uncertainty in model coefficients, variability in severity distributions and disability weights, and differences in sample size across data sources (15). To quantify this uncertainty in the final estimates, 1,000 draws were taken from the posterior distribution of the Bayesian meta-regression tool version 2.1 (DisMod-MR 2.1), and corresponding 95% uncertainty intervals (UIs) were generated in registered data. Specifically, prevalence in GBD was estimated using DisMod-MR 2.1 by integrating incidence data with disease duration, while YLDs was calculated by multiplying prevalence by the corresponding disability weight and adjusting for comorbidities to quantify the loss of healthy life years (13). Additionally, only the nonfatal burden of hip fracture was measured in GBD 2021; thus, this study reports incidence, prevalence, and YLDs but not cause-specific mortality or YLLs.

#### 2.2.3 Socio-demographic index

The Socio-demographic index (SDI), introduced in 2015 by the Institute for Health Metrics and Evaluation (IHME) at the University of Washington, serves as a key metric for linking social development to population health outcomes (15). It is derived as the geometric mean of three core indicators: the total fertility rates up to age 25 (TFU25), the average years of education among individuals aged 15 and older (EDU15+), and lagged per capita distributive income (LDI). An SDI score of 0 corresponds to the highest fertility rates, lowest educational attainment, and lowest income levels, while a score of 1 indicates the opposite. Based on these scores, countries are grouped into five categories: high SDI, medium-high SDI, medium SDI, medium-low SDI, and low SDI.

### 2.3 Estimated annual percentage change and percentage change

The estimated annual percentage change (EAPC) is a well-established and widely used indicator that has been extensively applied in previous studies to track temporal trends in measures such as incidence and prevalence (16). In this study, we estimated trends in FHFPW incidence, prevalence, and YLDs from 1990 to 2021 by fitting a regression model to the natural logarithm of rates over time, with the slope of the fitted line used to calculate the EAPC (17). A trend was considered increasing if the lower 95% confidence interval (95% CI) of the EAPC exceeded 0, decreasing if the upper 95% CI was below 0, and statistically insignificant if the 95% CI included 0.


y=α+βx+ε

EAPC=100×(exp(β)−1).

(x: year, y: natural logarithm of the rate (e.g., mortality, prevalence, morbidity, DALYs), α: intercept, β: slope, ε: random error).

### 2.4 Joinpoint regression modeling

We used Joinpoint regression modeling, a statistical method commonly used in epidemiology to assess time trends in disease prevalence and incidence. The optimal number of Joinpoints was determined using the Bayesian Information Criterion (BIC), allowing for up to three Joinpoints (18). Statistical significance was assessed with Monte Carlo permutation tests (*p* < 0.05), and model adequacy was evaluated through residual analysis and visual inspection for epidemiological plausibility (18). The default modeling method—the grid search method—calculates the annual percentage change (APC) and its 95% CI to describe trends over time. In addition, the average annual percentage change (AAPC) from 1990 to 2021 was calculated to provide an overall assessment of trends.

### 2.5 Statistical analyses

In this study, we described the absolute burden, incidence, prevalence, YLDs, and their corresponding EAPCs attributable to FHFPW, stratified by age group and region (13). Spearman’s correlation analysis was conducted to examine the associations between SDI and the FHFPW disease burden. Temporal trends were further evaluated using the Joinpoint regression to calculate the AAPC with 95% CIs.

### 2.6 Statistical software

All analyses were performed using R software (version 4.4.1).

## 3 Results

### 3.1 Global level

#### 3.1.1 Prevalence trends

**Figure 1:**
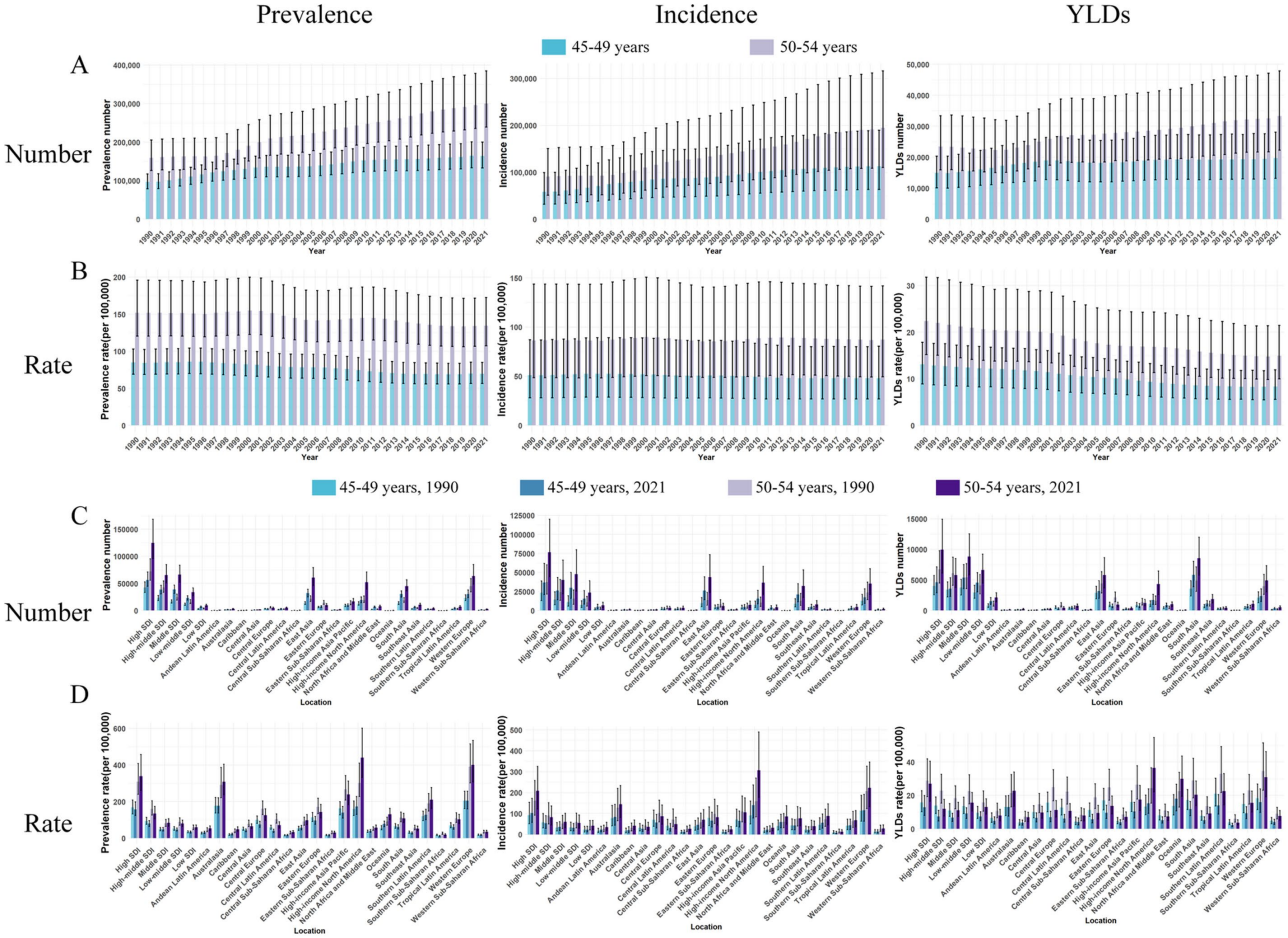
Prevalence of FHFPW by time and region, incidence, YLDs. **(A)** Case number of FHFPW for the 45-49 and 50-54 age groups for each year from 1900 through 2021. **(B)** Rates of FHFPW for the 45-49 and 50-54 age groups for each year from 1900 through 2021. **(C)** Case number of FHFPW for 2021 by region. **(D)** Case number of FHFPW for 2021 by FHFPW rates by region.

In 2021, the global prevalence of FHFPW in persons aged 45–49 years was 164,196 (95% UI: 132,953–199,964), up from 96,718 in 1990, representing a 69.8% increase (95% UI: 45.1–94.5%). However, the prevalence rate declined from 85.0 to 69.7 per 100,000, with an overall decrease of 18.0% (95% UI: −29.9 to −6.1%) and an EAPC of −0.9 (95% CI: −0.9 to −0.8). In the 50–54 age group, cases rose from 126,511 to 300,104 (88.2% increase; 95% UI: 54.9–121.4%), while the prevalence rate decreased from 152.0 to 134.6 per 100,000 (−11.5%; 95% UI: −27.1 to −4.2%) with an EAPC of −0.5 (95% CI: −0.6 to −0.4) (Table 1). From 1990 to 2021, prevalence and incidence numbers increased in both age groups, with a faster rise in the 50–54 group. YLD counts were stable in the 45–49 group but showed a gradual increase in the 50–54 group (Figure 1). Over the same period, prevalence rates declined slightly, incidence rates remained stable, and YLD rates decreased modestly in both groups (Figure 1). Overall, prevalence, incidence, and YLDs were consistently higher in the 50–54 age group (Supplementary Tables S1, S2).

**Table 1:** Prevalence of fall-induced hip fractures in perimenopausal women in 1990 and 2021 and change from 1990 to 2021.

location	Counts			Rate (per 100,000)			
	1990 (95% UI)	2021 (95% UI)	Percentage change (100, 95%UI)	1990 (95% UI)	2021 (95% UI)	Percentage change (100, 95%UI)	EAPC (100, 95% CI)
45–49 years							
Global	96718.0317 (78710.2224 to 117209.6248)	164195.9603 (132953.1521 to 199964.1838)	0.6977 (0.4508 to 0.9446)	84.989 (69.165 to 102.9955)	69.6801 (56.4215 to 84.8591)	−0.1801 (−0.2993 to −0.0609)	−0.85 (−0.93 to −0.77)
High SDI	42455.7086 (33544.9837 to 52874.3024)	56483.1484 (44736.1024 to 71518.1563)	0.3304 (0.1073 to 0.5535)	168.0307 (132.7639 to 209.2652)	157.3082 (124.5921 to 199.1813)	−0.0638 (−0.2208 to 0.0932)	−0.14 (−0.22 to −0.06)
High-middle SDI	23136.2181 (18608.0001 to 27974.5084)	38476.2615 (31004.1148 to 46526.8777)	0.663 (0.4205 to 0.9055)	94.3665 (75.8971 to 114.1007)	79.7799 (64.2865 to 96.4727)	−0.1546 (−0.2779 to −0.0313)	−0.86 (−0.99 to −0.74)
Middle SDI	16590.3525 (13612.684 to 19752.1038)	38875.264 (31586.3389 to 47035.5179)	1.3432 (1.0186 to 1.6678)	48.9492 (40.1637 to 58.2778)	47.9284 (38.9421 to 57.989)	−0.0209 (−0.1565 to 0.1147)	−0.42 (−0.69 to −0.15)
Low-middle SDI	11540.1398 (9518.1212 to 13737.9721)	23215.3253 (19225.4967 to 27490.7188)	1.0117 (0.7498 to 1.2736)	53.1678 (43.8519 to 63.2936)	46.9594 (38.8888 to 55.6075)	−0.1168 (−0.2318 to −0.0018)	−0.52 (−0.56 to −0.48)
Low SDI	2906.1628 (2405.1247 to 3413.9673)	7032.5912 (5841.9779 to 8239.441)	1.4199 (1.1195 to 1.7203)	35.007 (28.9716 to 41.1239)	33.8636 (28.1305 to 39.6748)	−0.0327 (−0.1528 to 0.0874)	−0.19 (−0.24 to −0.13)
Andean Latin America	195.1343 (159.4522 to 235.7778)	589.375 (473.4767 to 716.7254)	2.0204 (1.5823 to 2.4585)	27.4517 (22.4319 to 33.1695)	31.5057 (25.3102 to 38.3133)	0.1477 (−0.0188 to 0.3142)	0.42 (0.31–0.53)
Australasia	1001.7973 (781.35 to 1259.5375)	1786.2998 (1385.5848 to 2232.7601)	0.7831 (0.477 to 1.0892)	176.9472 (138.0097 to 222.4718)	177.2496 (137.4877 to 221.5506)	0.0017 (−0.1702 to 0.1736)	0.11 (−0.03–0.25)
Caribbean	156.658 (124.1644 to 195.2141)	338.2009 (267.2573 to 416.3451)	1.1588 (0.8105 to 1.5071)	19.8369 (15.7224 to 24.7191)	23.2947 (18.4083 to 28.6772)	0.1743 (−0.0152 to 0.3638)	0.6 (0.42–0.77)
Central Asia	602.3553 (500.6096 to 710.2841)	1176.3701 (975.1402 to 1398.4781)	0.953 (0.7036 to 1.2024)	51.4782 (42.7829 to 60.702)	42.8282 (35.502 to 50.9145)	−0.168 (−0.2743 to −0.0617)	−0.5 (−0.57 to −0.44)
Central Europe	3419.175 (2776.3985 to 4134.4284)	3219.4694 (2563.9948 to 3986.2391)	−0.0584 (−0.2011 to 0.0843)	100.2973 (81.4422 to 121.2784)	75.1442 (59.8451 to 93.0411)	−0.2508 (−0.3643 to −0.1373)	−1.03 (−1.13 to −0.94)
Central Latin America	1768.7712 (1468.7425 to 2121.3756)	3193.9725 (2565.3495 to 3892.2725)	0.8058 (0.5498 to 1.0618)	59.0772 (49.0562 to 70.8542)	40.2734 (32.3469 to 49.0783)	−0.3183 (−0.4149 to −0.2217)	−0.63 (−0.88 to −0.39)
Central Sub-Saharan Africa	138.2367 (111.695 to 173.0214)	420.5468 (340.239 to 515.0849)	2.0422 (1.5703 to 2.5141)	15.3828 (12.4293 to 19.2536)	16.929 (13.6962 to 20.7346)	0.1005 (−0.0702 to 0.2712)	0.4 (0.31–0.5)
East Asia	13636.596 (11036.7877 to 16525.5393)	31908.7706 (25497.2603 to 39220.8338)	1.3399 (0.9883 to 1.6915)	53.5594 (43.3483 to 64.9061)	56.8495 (45.4266 to 69.8769)	0.0614 (−0.0981 to 0.2209)	−0.54 (−1.19–0.11)
Eastern Europe	6437.3816 (5169.9683 to 7955.5803)	7269.9729 (5756.5847 to 8981.1184)	0.1293 (−0.0492 to 0.3078)	114.107 (91.6412 to 141.0181)	96.0554 (76.0595 to 118.6641)	−0.1582 (−0.2913 to −0.0251)	−0.82 (−1.21 to −0.43)
Eastern Sub-Saharan Africa	489.6976 (398.3337 to 594.8234)	1244.0703 (1017.4914 to 1509.4684)	1.5405 (1.1754 to 1.9056)	16.9998 (13.8281 to 20.6493)	16.5086 (13.502 to 20.0304)	−0.0289 (−0.1685 to 0.1107)	0.02 (−0.08–0.12)
High-income Asia Pacific	9448.5721 (7416.6799 to 11580.093)	10003.6104 (7901.7901 to 12456.6011)	0.0587 (−0.1124 to 0.2298)	162.8628 (127.8395 to 199.6033)	139.4641 (110.1618 to 173.6621)	−0.1437 (−0.2821 to −0.0053)	−0.51 (−0.69 to −0.32)
High-income North America	12664.6867 (9819.1206 to 16152.9141)	19410.8137 (14615.8014 to 25444.4106)	0.5327 (0.2398 to 0.8256)	159.8978 (123.9712 to 203.9384)	173.759 (130.8357 to 227.7697)	0.0867 (−0.121 to 0.2944)	0.29 (0.2–0.38)
North Africa and Middle East	2161.7319 (1808.1308 to 2590.3781)	6762.2536 (5563.0123 to 8026.7314)	2.1282 (1.7184 to 2.538)	37.4123 (31.2926 to 44.8307)	40.0459 (32.944 to 47.5341)	0.0704 (−0.0698 to 0.2106)	0.05 (−0.07–0.18)
Oceania	63.8133 (52.5465 to 78.4582)	252.0287 (205.5098 to 303.132)	2.9495 (2.3841 to 3.5149)	55.5601 (45.7505 to 68.3109)	77.949 (63.5614 to 93.7546)	0.403 (0.2022 to 0.6038)	1.02 (0.97–1.08)
South Asia	13758.0801 (11252.1666 to 16442.7257)	30407.9656 (24915.0175 to 36337.1489)	1.2102 (0.91 to 1.5104)	66.378 (54.2878 to 79.3304)	61.7181 (50.5692 to 73.7524)	−0.0702 (−0.1965 to 0.0561)	−0.35 (−0.39 to −0.32)
Southeast Asia	3006.8591 (2468.6394 to 3593.7905)	6454.668 (5221.7113 to 7798.102)	1.1466 (0.847 to 1.4462)	32.6383 (26.7961 to 39.0092)	28.959 (23.4273 to 34.9864)	−0.1127 (−0.2365 to 0.0111)	−0.59 (−0.66 to −0.53)
Southern Latin America	1537.7975 (1233.0663 to 1902.0859)	2783.6834 (2223.2028 to 3463.1726)	0.8102 (0.5227 to 1.0977)	119.7932 (96.0549 to 148.171)	127.3557 (101.7133 to 158.4429)	0.0631 (−0.1058 to 0.232)	0.22 (−0.01–0.45)
Southern Sub-Saharan Africa	167.5185 (135.2589 to 206.8413)	244.9821 (197.9613 to 311.9625)	0.4624 (0.2267 to 0.6981)	17.6013 (14.2118 to 21.733)	11.6578 (9.4202 to 14.8451)	−0.3377 (−0.4444 to −0.231)	−1.33 (−1.62 to −1.05)
Tropical Latin America	2233.709 (1823.6545 to 2696.3603)	4596.2349 (3722.7703 to 5511.5159)	1.0577 (0.7682 to 1.3472)	70.0438 (57.1854 to 84.5514)	61.0684 (49.463 to 73.2294)	−0.1281 (−0.2508 to −0.0054)	−0.62 (−0.91 to −0.32)
Western Europe	23277.8524 (18315.5995 to 29098.9349)	30528.3925 (24082.9651 to 38234.8301)	0.3115 (0.0922 to 0.5308)	205.2822 (161.5212 to 256.6171)	204.6489 (161.4416 to 256.3095)	−0.0031 (−0.1698 to 0.1636)	0.05 (−0.1–0.21)
Western Sub-Saharan Africa	551.6083 (448.902 to 664.7902)	1604.2791 (1307.5252 to 1958.8682)	1.9084 (1.49 to 2.3268)	18.6302 (15.1614 to 22.4529)	18.2499 (14.8741 to 22.2836)	−0.0204 (−0.1613 to 0.1205)	0.02 (−0.07–0.1)
50–54 years							
Global	159493.0744 (126511.2997 to 205436.0148)	300104.3675 (239225.5038 to 384914.339)	0.8816 (0.5489 to 1.2143)	152.0186 (120.5825 to 195.8085)	134.6106 (107.3036 to 172.6518)	−0.1145 (−0.2711 to 0.0421)	−0.48 (−0.55 to −0.4)
High SDI	72238.8488 (56209.2331 to 95515.4585)	124709.9456 (96243.9541 to 168497.5219)	0.7264 (0.3764 to 1.0764)	309.2583 (240.6346 to 408.9066)	338.8043 (261.4697 to 457.7637)	0.0955 (−0.1266 to 0.3176)	0.39 (0.34–0.43)
High-middle SDI	42741.7314 (33874.5629 to 54885.8815)	65210.7626 (51310.2713 to 84882.0539)	0.5257 (0.2487 to 0.8027)	159.8346 (126.6754 to 205.2481)	134.5322 (105.855 to 175.1148)	−0.1583 (−0.3111 to −0.0055)	−0.77 (−0.88 to −0.66)
Middle SDI	24239.0056 (19223.5733 to 30581.7624)	66395.3141 (52447.868 to 83932.4729)	1.7392 (1.2734 to 2.205)	81.6337 (64.7424 to 102.9953)	84.1326 (66.4592 to 106.3548)	0.0306 (−0.1447 to 0.2059)	−0.16 (−0.42–0.1)
Low-middle SDI	16106.8581 (12845.5489 to 20219.3634)	33898.8775 (27551.5763 to 42013.008)	1.1046 (0.7686 to 1.4406)	88.8096 (70.8274 to 111.485)	80.1561 (65.1475 to 99.3425)	−0.0974 (−0.2415 to 0.0467)	−0.42 (−0.45 to −0.39)
Low SDI	4013.0391 (3245.4425 to 4929.4219)	9698.7512 (7975.1402 to 11694.272)	1.4168 (1.0663 to 1.7673)	58.3629 (47.1995 to 71.6901)	59.588 (48.9983 to 71.8482)	0.021 (−0.1271 to 0.1691)	0.05 (0–0.09)
Andean Latin America	260.6159 (209.8734 to 328.9185)	863.8066 (669.2505 to 1081.1986)	2.3145 (1.7561 to 2.8729)	44.9921 (36.232 to 56.7837)	53.6335 (41.5536 to 67.1313)	0.1921 (−0.0087 to 0.3929)	0.5 (0.39–0.62)
Australasia	1373.3938 (1046.5742 to 1829.5466)	3090.5346 (2378.1417 to 4040.6912)	1.2503 (0.8003 to 1.7003)	290.9491 (221.7134 to 387.5835)	309.2094 (237.9342 to 404.273)	0.0628 (−0.1497 to 0.2753)	0.33 (0.21–0.45)
Caribbean	260.3371 (204.5403 to 335.7952)	679.1561 (529.4355 to 863.3727)	1.6088 (1.1401 to 2.0775)	39.2791 (30.8606 to 50.6641)	48.445 (37.7652 to 61.5854)	0.2334 (0.0118 to 0.455)	0.63 (0.46–0.79)
Central Asia	1206.902 (973.1001 to 1522.3591)	1613.0272 (1314.5173 to 1957.3661)	0.3365 (0.1303 to 0.5427)	78.5396 (63.3248 to 99.068)	64.3012 (52.4015 to 78.0278)	−0.1813 (−0.3076 to −0.055)	−0.53 (−0.61 to −0.45)
Central Europe	5826.3984 (4678.7476 to 7382.6226)	4916.3055 (3815.9951 to 6318.2697)	−0.1562 (−0.3045 to −0.0079)	161.3448 (129.564 to 204.4398)	125.5703 (97.4666 to 161.3786)	−0.2217 (−0.3584 to −0.085)	−1 (−1.11 to −0.89)
Central Latin America	2564.3706 (2040.1182 to 3266.8454)	5061.623 (4003.1381 to 6459.9808)	0.9738 (0.6306 to 1.317)	104.1123 (82.8279 to 132.6325)	70.6103 (55.8443 to 90.1176)	−0.3218 (−0.4397 to −0.2039)	−0.79 (−1.01 to −0.57)
Central Sub-Saharan Africa	238.0786 (185.9986 to 303.2233)	676.2326 (536.5976 to 847.0708)	1.8404 (1.3526 to 2.3282)	29.9713 (23.415 to 38.1722)	33.5842 (26.6494 to 42.0686)	0.1205 (−0.0719 to 0.3129)	0.42 (0.34–0.51)
East Asia	21326.667 (16680.236 to 27216.9384)	60530.6249 (46366.9978 to 79404.8125)	1.8383 (1.3053 to 2.3713)	90.689 (70.9306 to 115.7366)	98.0809 (75.1309 to 128.6637)	0.0815 (−0.1216 to 0.2846)	−0.16 (−0.66–0.35)
Eastern Europe	14498.8326 (11449.2544 to 18667.5376)	9882.367 (7717.8219 to 12750.1771)	−0.3184 (−0.4422 to −0.1946)	170.5525 (134.6797 to 219.5897)	142.8491 (111.5607 to 184.3032)	−0.1624 (−0.3146 to −0.0102)	−0.91 (−1.32 to −0.51)
Eastern Sub-Saharan Africa	737.21 (580.6609 to 922.1896)	1749.2567 (1386.5794 to 2134.5879)	1.3728 (0.9912 to 1.7544)	31.1299 (24.5194 to 38.941)	30.8382 (24.4444 to 37.6313)	−0.0094 (−0.1687 to 0.1499)	0.08 (−0.03–0.19)
High-income Asia Pacific	13984.4379 (10953.0721 to 17963.0875)	16957.9422 (12969.6512 to 22157.6873)	0.2126 (−0.0157 to 0.4409)	266.5215 (208.7484 to 342.3483)	239.3529 (183.0602 to 312.7447)	−0.1019 (−0.271 to 0.0672)	−0.32 (−0.48 to −0.16)
High-income North America	19779.1457 (14946.3815 to 26913.1311)	52112.7419 (39391.8205 to 71445.4175)	1.6347 (1.0548 to 2.2146)	302.1573 (228.3293 to 411.1401)	439.6688 (332.3439 to 602.7761)	0.4551 (0.1348 to 0.7754)	1.43 (1.28–1.58)
North Africa and Middle East	2574.7729 (2120.3213 to 3106.4956)	7951.798 (6469.2707 to 9901.8641)	2.0883 (1.6336 to 2.543)	51.7421 (42.6095 to 62.4275)	57.6225 (46.8794 to 71.7536)	0.1136 (−0.0504 to 0.2776)	0.21 (0.08–0.33)
Oceania	84.4777 (66.0091 to 106.1616)	334.2577 (265.1661 to 419.3843)	2.9568 (2.2882 to 3.6254)	91.6876 (71.6428 to 115.2222)	130.2007 (103.288 to 163.3593)	0.42 (0.18 to 0.66)	1.04 (0.98–1.1)
South Asia	19046.8631 (15140.073 to 24212.296)	45121.4935 (36214.8006 to 56854.8451)	1.369 (0.9699 to 1.7681)	112.8553 (89.707 to 143.4612)	107.5696 (86.336 to 135.5419)	−0.0468 (−0.2074 to 0.1138)	−0.23 (−0.27 to −0.2)
Southeast Asia	4706.1703 (3748.614 to 5854.6662)	10755.5304 (8615.0905 to 13466.4705)	1.2854 (0.915 to 1.6558)	57.616 (45.8929 to 71.6766)	52.629 (42.1554 to 65.8942)	−0.0866 (−0.2347 to 0.0615)	−0.54 (−0.62 to −0.47)
Southern Latin America	2151.4935 (1684.8041 to 2770.3095)	4122.0184 (3196.4455 to 5421.739)	0.9159 (0.5548 to 1.277)	189.5598 (148.4416 to 244.0812)	210.289 (163.0699 to 276.5955)	0.1094 (−0.0997 to 0.3185)	0.38 (0.15–0.61)
Southern Sub-Saharan Africa	223.172 (177.849 to 277.2732)	337.1607 (265.1533 to 421.9308)	0.5108 (0.2626 to 0.759)	27.0521 (21.5582 to 33.6101)	19.0051 (14.9462 to 23.7834)	−0.2975 (−0.4129 to −0.1821)	−1.12 (−1.38 to −0.85)
Tropical Latin America	2945.5563 (2325.3811 to 3751.4704)	7070.7011 (5599.6728 to 8971.2339)	1.4005 (0.9844 to 1.8166)	109.0872 (86.1193 to 138.9338)	103.0176 (81.5853 to 130.7077)	−0.0556 (−0.2193 to 0.1081)	−0.36 (−0.64 to −0.08)
Western Europe	44879.6519 (34794.8767 to 59204.7176)	63776.0598 (49036.1017 to 85079.1557)	0.421 (0.1367 to 0.7053)	390.9265 (303.0825 to 515.7057)	401.211 (308.4829 to 535.2274)	0.0263 (−0.1791 to 0.2317)	0.14 (0–0.28)
Western Sub-Saharan Africa	824.5272 (659.661 to 1026.7611)	2501.7295 (1990.857 to 3103.8944)	2.0341 (1.5469 to 2.5213)	34.8677 (27.8958 to 43.4197)	34.9951 (27.8489 to 43.4185)	0.0037 (−0.1575 to 0.1649)	0.08 (0–0.16)

### 3.2 Regional level

#### 3.2.1 Prevalence trends

**Figure 2:**
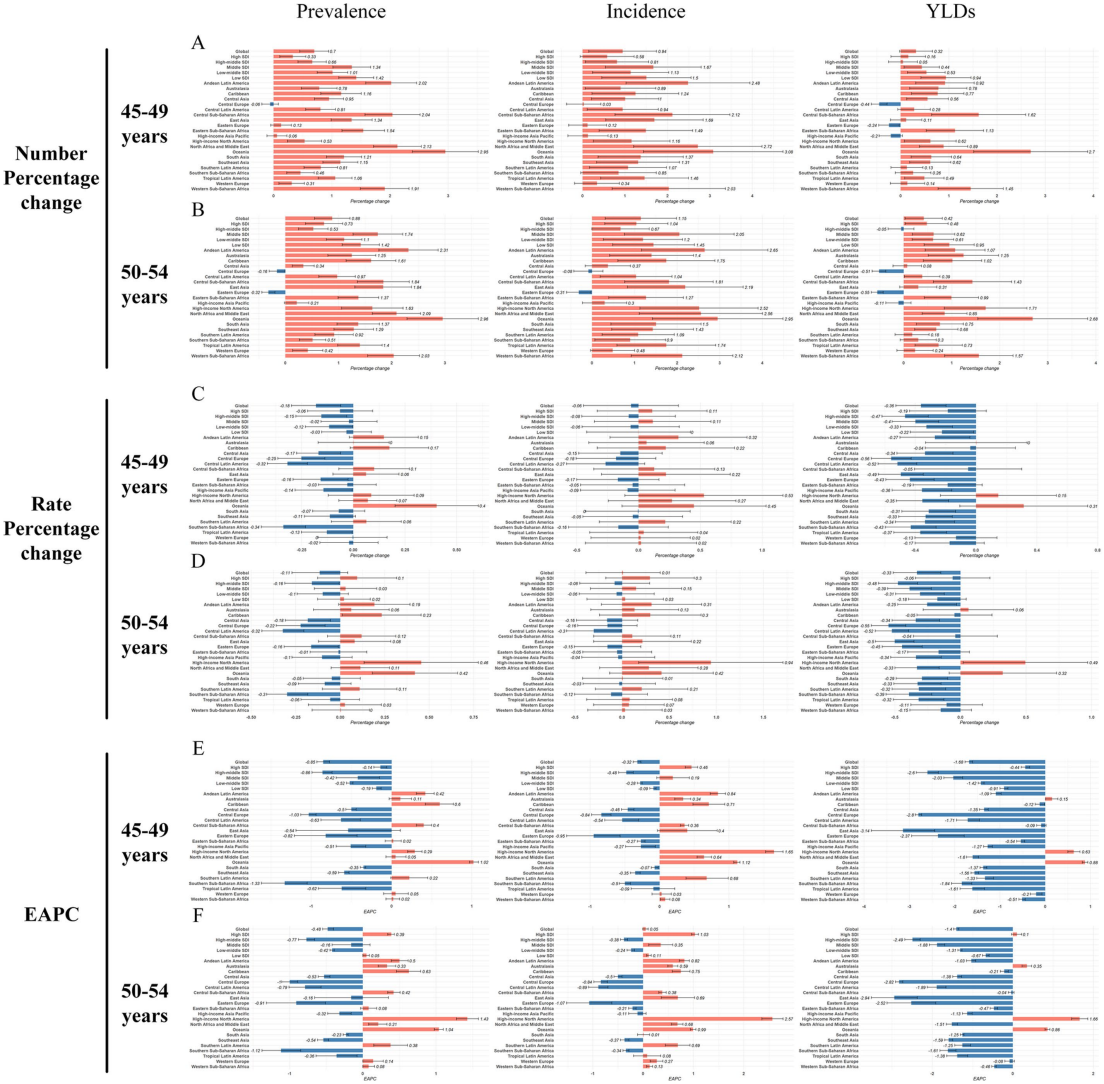
Changes in the burden of disease for FHFPW globally and by region. **(A,B)** Number percentage change in FHFPW from 1990 to 2021. **(C,D)** Rate percentage change in FHFPW from 1990 to 2021. **(E,F)** EAPC for FHFPW from 1990 to 2021.

The global burden of FHFPW varied substantially across regions and was strongly associated with SDI. In the 45–49 age group, the High SDI region had the highest prevalence (56,483; 95% UI: 44,736–71,518), while Oceania showed the largest relative increase (295.0%). Similarly, in the 50–54 age group, the High SDI region reported the highest prevalence (124,710; 95% UI: 96,244–168,498), with Oceania again exhibiting the greatest relative increase (295.7%) (Table 1; Figure 2). Other regions with high prevalence in 2021 included East Asia, High-income North America, South Asia, and Western Europe (Table 1; Figure 1). In the 45–49 age group, Western Europe had the highest prevalence (204.7 per 100,000), while Oceania showed the greatest relative increase (40.3%) and highest EAPC (1.0; 95% CI: 1.0–1.1). For the 50–54 age group, High-income North America reported the highest prevalence (439.67 per 100,000) and the largest increase (EAPC = 1.4; 95% CI: 1.3–1.6). Other regions with high prevalence included Western Europe, Australasia, and High-income Asia Pacific (Table 1; Figures 1, 2).

#### 3.2.2 Incidence patterns

Incidence cases varied markedly by region and age group. In the 45–49 age group, the High SDI region reported the highest number of cases (36,924; 95% UI: 20,921–62,197), while Oceania showed the largest relative increase (307.8%). For the 50–54 age group, cases again peaked in the High SDI region (76,550), with Oceania showing the greatest increase (295.2%; 95% UI: 141.8–448.6). Other regions with high case numbers included East Asia, High-income North America, South Asia, and Western Europe (32,212–43,799) (Supplementary Table S1; Figures 1, 2). High-income North America consistently had the highest incidence rates, reaching 140.5 per 100,000 in the 45–49 age group (relative increase: 52.9%; EAPC = 1.7, 95% CI: 1.5–1.8) and 307.3 per 100,000 in the 50–54 age group (increase: 94.1%; EAPC = 2.6, 95% CI: 2.4–2.8). Other regions with high incidence rates included Australasia and Western Europe (144.7–222.6 per 100,000) (Supplementary Table S1; Figure 1).

#### 3.2.3 YLDs patterns

For YLD counts, South Asia recorded the highest in the 45–49 age group (5,821; 95% UI: 3,890–8,056), while the High SDI region led in the 50–54 age group (9,971; 95% UI: 6,146–14,935). Oceania showed the largest relative increase in both groups (>260%). East Asia, High-income North America, and Western Europe also contributed substantially, with 2021 YLDs ranging from 4,314 to 5,792 (Supplementary Table S2; Figure 1). For YLD rates, Oceania was highest in the 45–49 age group (18.07 per 100,000; EAPC = 0.9, 95% CI: 0.8–0.9), whereas High-income North America ranked first in the 50–54 group (36.4 per 100,000; EAPC = 1.7, 95% CI: 1.5–1.9). Other regions with high rates in 2021 included Western Europe, Australasia, and Southern Latin America (22.5–31.0 per 100,000) (Supplementary Table S2; Figure 2).

### 3.3 SDI level

#### 3.3.1 Spearman’s correlation between FHFPW burden and SDI

**Figure 3:**
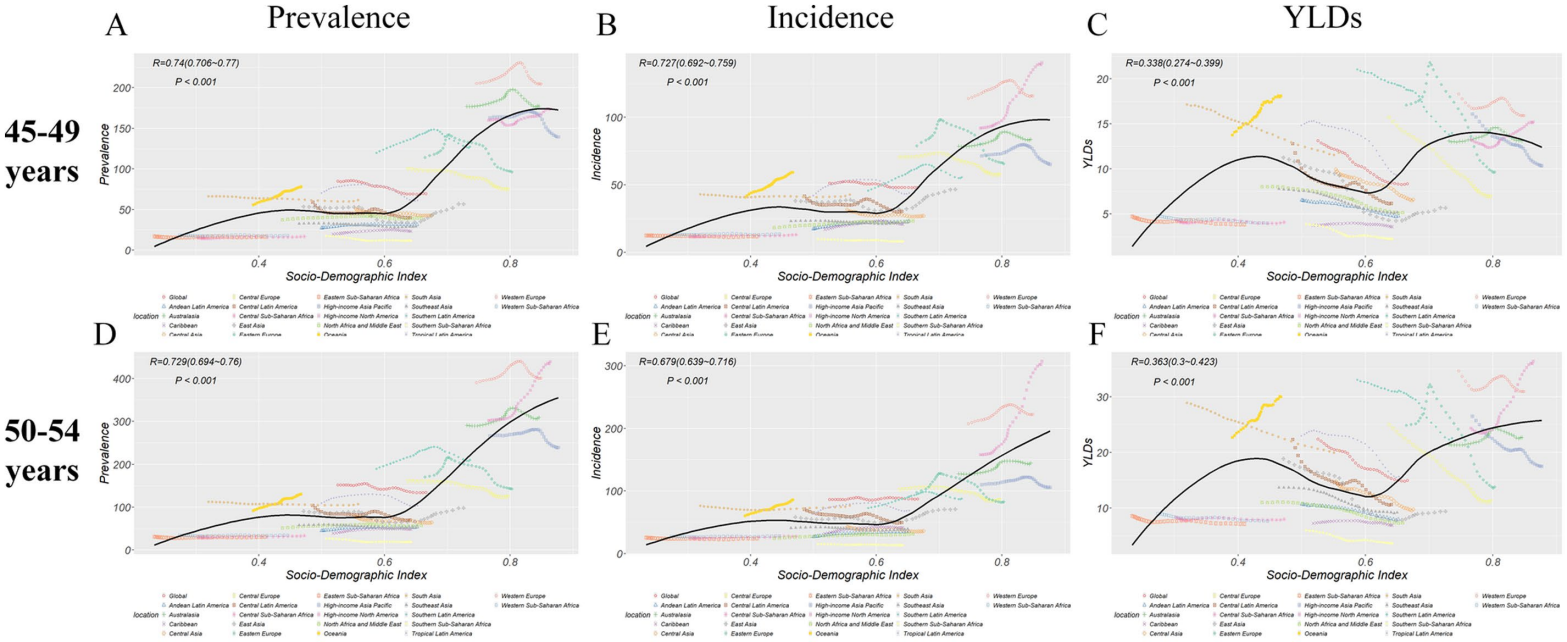
Correlation analysis between SDI and rates. **(A-C)** Correlation analysis in age group 45-49 years between FHFPW rate and SDI. **(D-F)** Correlation analysis in age group 50-54 years between FHFPW rate and SDI.

Spearman’s rank-order analyses showed that FHFPW burden was positively associated with SDI. In both the 45–49 and 50–54 age groups, prevalence, incidence, and YLD numbers exhibited only weak positive correlations (all *p* < 0.001). By contrast, rates showed stronger associations: prevalence rates had the strongest positive correlation with SDI (*p* < 0.001), while YLD rates demonstrated weaker but still significant positive correlations (*p* < 0.001) (Figure 3; Supplementary Figure S1).

#### 3.3.2 Prevalence trends by SDI region

**Figure 4:**
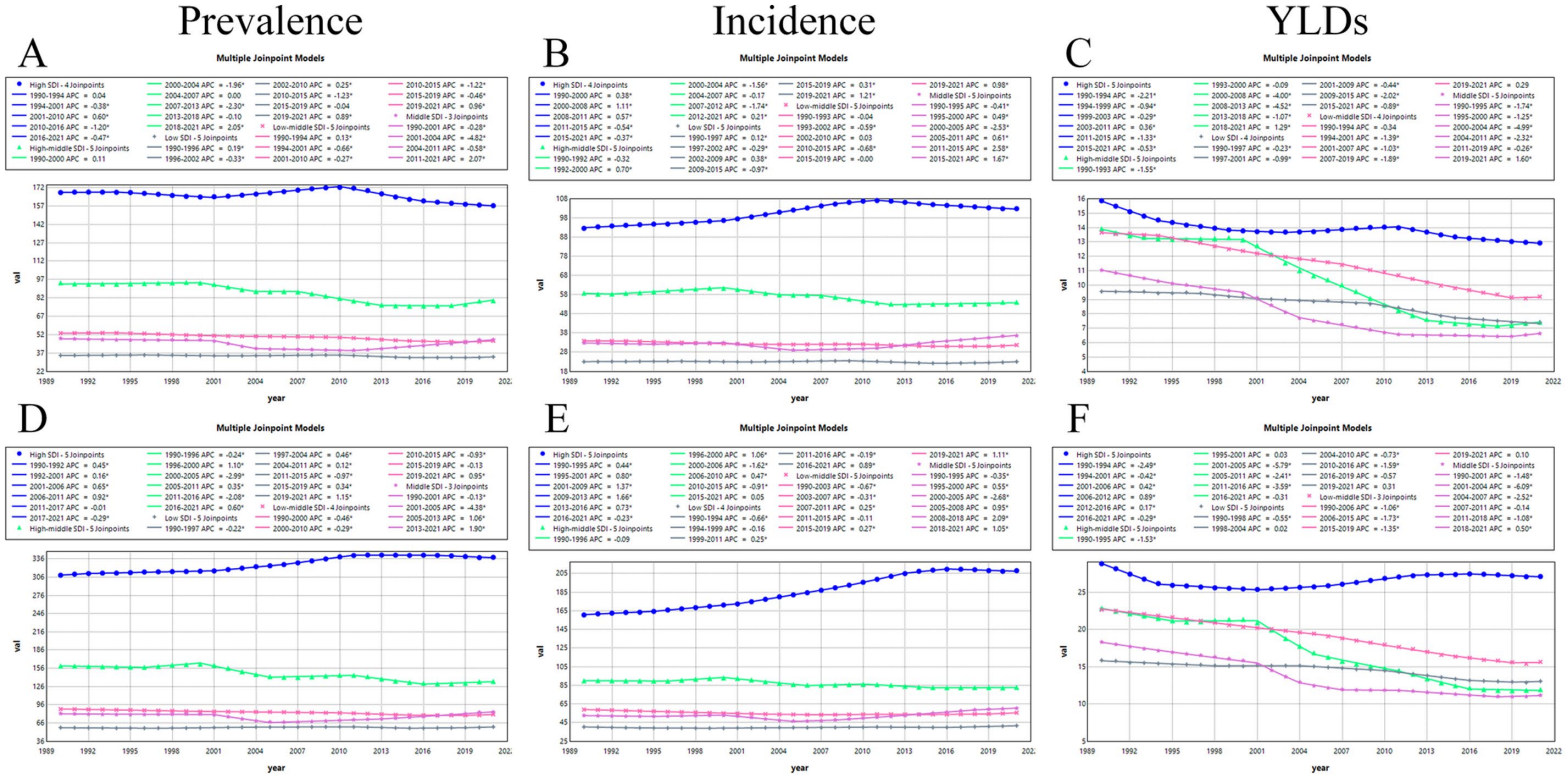
Joinpoint analysis. **(A-C)** Joinpoint analysis of prevalence, incidence and YLDs in the age group 45-49 years. **(D-F)** Joinpoint analysis of prevalence, incidence and YLDs in the age group 50-54 years.

Trends in FHFPW from 1990 to 2021 across SDI levels were analyzed using Joinpoint regression. In the 45–49 age group, prevalence rates declined across all SDI regions, with the largest decrease in the High-middle SDI region (AAPC = −0.5%), most notable from 2007 to 2013 (AAPC = −2.3%) and followed by an increase from 2018 to 2021 (AAPC = 2.1%). The Middle SDI region showed the smallest overall decline (AAPC = −0.05%), with a sharp fall from 2001 to 2004 (AAPC = −4.8%) and an increase from 2011 to 2021 (AAPC = 2.1%) (Figure 4; Supplementary File 1). Incidence rates increased in High SDI (AAPC = 0.3%) and Middle SDI regions (AAPC = 0.4%) (Figure 4, Supplementary File 2). YLD rates decreased across all regions, most markedly in the High-middle SDI region (AAPC = −2.0%) and least in the High SDI region (AAPC = −0.7%) (Figure 4; Supplementary File 3). In the 50–54 age group, prevalence increased most in the High SDI region (AAPC = 0.3%), particularly from 2006 to 2011 (APC = 0.9%), then stabilized during 2011–2017 (APC = −0.01%) before declining in 2017–2021 (APC = −0.3%). Middle and Low SDI regions also showed upward prevalence trends, with AAPCs of 0.1 and 0.07%, respectively (Figure 4; Supplementary File 4).

#### 3.3.3 Incidence trends by SDI region

For incidence rates, the strongest increase occurred in the High SDI region (AAPC = 0.82%), followed by the Middle SDI region (AAPC = 0.8%). The smallest increase was observed in the Low SDI region (AAPC = 0.1%). In contrast, both High-middle and Low-middle SDI regions showed declines, with AAPCs of −0.3% and −0.2%, respectively (Figure 4; Supplementary File 5).

#### 3.3.4 YLD rate trends by SDI region

For YLD rates, all SDI regions showed decreasing trends from 1990 to 2021. The largest decline occurred in the High-middle SDI region (AAPC = −2.1%), while the smallest decline was observed in the High SDI region (AAPC = −0.2%). Notably, the High SDI region exhibited temporary upward trends between 2001 and 2006 (AAPC = 0.4), 2006–2012 (AAPC = 0.9%), and 2012–2016 (AAPC = 0.2%) (Figure 4; Supplementary File 6).

### 3.4 National level

#### 3.4.1 Prevalence trends

**Figure 5:**
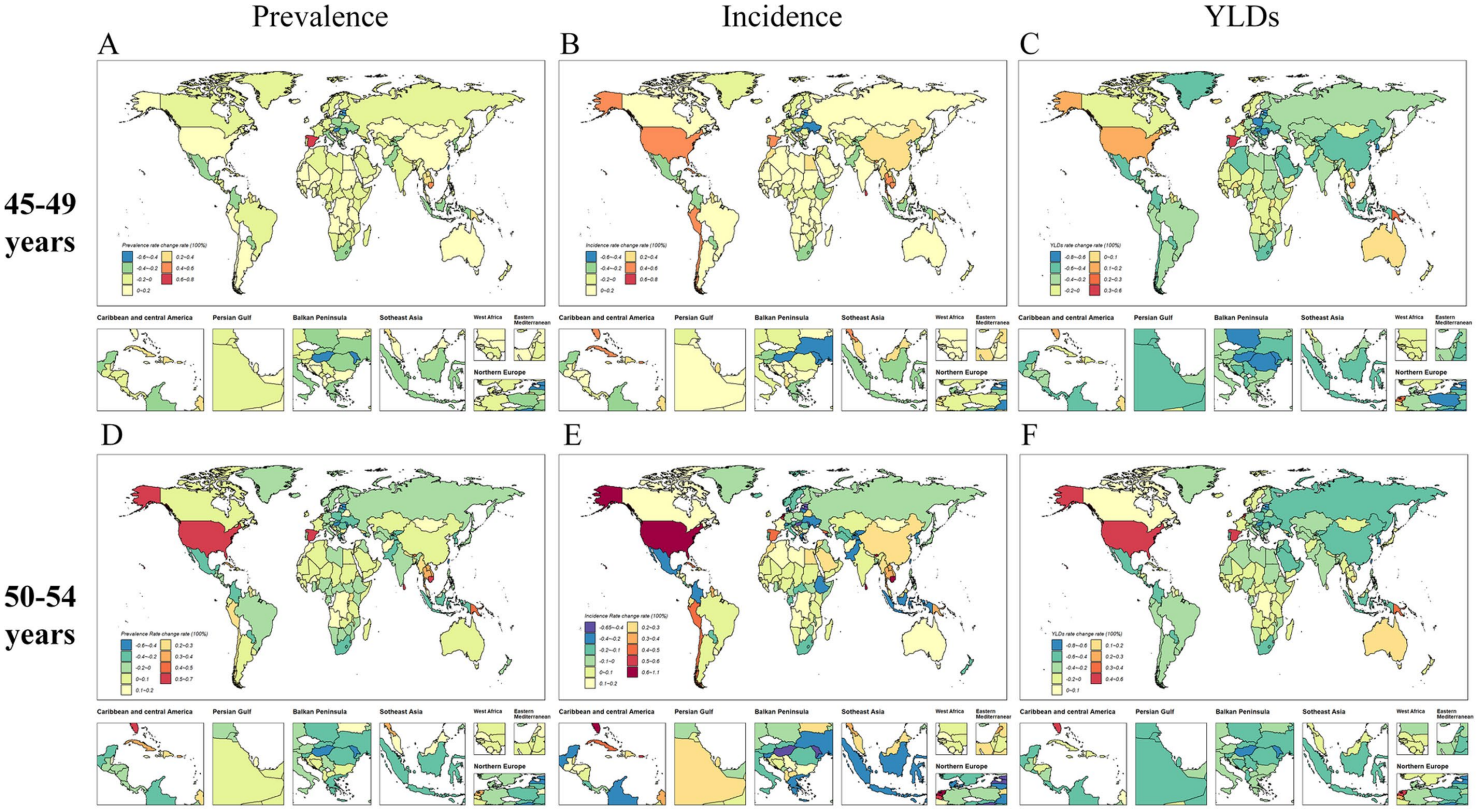
Global distribution of the rates of change in FHFPW disease burden from 1990 to 2021. **(A-C)** Global distribution of the rate of change in FHFPW disease burden rate in the 45-49 age group. **(D-F)** Global distribution of the rate of change in FHFPW disease burden rate in the 50-54 age group.

At the national level, country-specific distributions of FHFPW prevalence from 1990 to 2021 were shown in Figure 5 and Supplementary Figure S2. In the 45–49 age group, the largest increases in prevalence number were observed in the United Arab Emirates (12.0 per 100,000), Qatar (10.5), Bahrain (5.4), Kuwait (5.1), and Jordan (4.6). In the 50–54 age group, the highest increases were in the United Arab Emirates (12.2), Qatar (11.4), Kuwait (6.1), Bahrain (5.3), and Jordan (4.5) (Supplementary Files 7, 8). For prevalence rates, the greatest increases in the 45–49 age group occurred in Spain (0.7), Cambodia (0.5), Papua New Guinea (0.4), Sri Lanka (0.4), and Cuba (0.4). In the 50–54 age group, the largest increases were seen in Spain (0.6), Cambodia (0.5), Sri Lanka (0.5), and the United States (0.50) (Supplementary Files 9, 10).

#### 3.4.2 Incidence patterns

Regarding incidence cases, several countries showed high growth from 1990 to 2021(Figure 5; Supplementary Figure S2). In the 45–49 age group, the largest increases occurred in the United Arab Emirates (12.9), Puerto Rico (12.6), Kiribati (6.6), Bahamas (6.5), and Sao Tome and Principe (5.2). In the 50–54 age group, notable increases were observed in Qatar (12.7), United Arab Emirates (12.1), Kuwait (7.5), Bahrain (5.9), and Saudi Arabia (5.1) (Supplementary Files 11, 12). For incidence rates, the highest increases in the 45–49 age group were in Sri Lanka (0.7), Bhutan (0.6), the United States (0.6), Spain (0.6), and Cambodia (0.55). In the 50–54 age group, the largest increases were seen in the United States (1.0), Sri Lanka (0.8), Bhutan (0.7), Cambodia (0.6), and the Netherlands (0.6) (Supplementary Files 13, 14).

#### 3.4.3 YLDs patterns

The number of YLDs also showed marked changes from 1990 to 2021(Figure 5; Supplementary Figure S2). In the 45–49 age group, the largest increases occurred in the United Arab Emirates (7.2), Qatar (4.5), Belize (3.4), Papua New Guinea (3.3), and Djibouti (2.9). In the 50–54 age group, notable increases were observed in the United Arab Emirates (7.3), Qatar (4.7), Kuwait (3.4), Papua New Guinea (3.3), and Belize (3.2) (Supplementary Files 15, 16). For YLD rates, the 45–49 age group showed the largest increases in Spain (0.5), Papua New Guinea (0.3), the Netherlands (0.2), the United States (0.2), and Andorra (0.1). In the 50–54 age group, the highest increases were observed in the United States (0.5), Spain (0.4), the Netherlands (0.4), Papua New Guinea (0.3), and Belgium (0.3) (Supplementary Files 17, 18).

## 4 Discussion

This study provides the first comprehensive assessment of the disease burden of FHFPW and yielded several key findings: (1) the burden was higher in the 50–54 than in the 45–49 age group, with overall prevalence, incidence, and YLDs cases increasing from 1990 to 2021 despite declining rates; (2) Disease burden correlated positively with SDI, particular in the 45–49 age and Oceania (3) Joinpoint analysis revealed the most pronounced rise in incidence rate in the 50–54 age group within high-SDI regions (AAPC = 0.8%; 95% CI: 0.8–0.9; *p* < 0.001); (4) Differences in the burden of FHFPW were also observed among countries within the same SDI region. Collectively, these findings provide an evidence base to guide health policy, resource allocation, and the development of targeted prevention strategies for FHFPW.

From 1990 to 2021, the absolute burden of hip fractures in women aged 45–54 years increased, whereas the corresponding prevalence and YLDs rates declined. This declining trend may partly be attributable to the rapid expansion of the population base, greater public awareness of fracture prevention, and decades of accumulated clinical expertise that have shortened treatment and recovery times for new cases, collectively indicating progress in diagnosis, prevention, treatment, and prognosis (19, 20). In addition, the reduced risk of hip fracture may be associated with declines in smoking and alcohol consumption rates, as well as with global warming (21, 22). These findings highlight underscore the importance of continued public health measures and clinical advancements in mitigating the burden of hip fractures.

Our study confirmed a higher burden of FHFPW in high-SDI regions, consistent with previous findings (13, 23–26). Joinpoint analysis further supported this pattern, revealing the most pronounced rise in incidence in high-SDI regions. This may reflect rapid urbanization and industrialization in economically developed areas, which drive lifestyle changes such as sedentary behavior, psychosocial stress, and reduced physical activity (27–29). These explanations remain speculative and warrant confirmation in future studies. Notably, regional differences were observed within high-SDI areas: in the 45–49 age group, prevalence and YLD rates were highest in Western Europe, whereas in the 50–54 age group, both prevalence, incidence, and YLD rates peaked in high-income North America. These findings suggest that, even at similar SDI levels, the age distribution of FHFPW burden differs across regions, highlighting the need for region-specific preventive strategies targeting peri-menopausal women aged 45–54.

This study found that over the past 32 years, Oceania experienced the most pronounced increases in FHFPW prevalence, incidence, and YLDs, far exceeding global levels, with YLDs in the 45–49 age group reaching 8.44 times the global average (30). In addition to the psychosocial stress commonly faced by peri-menopausal women, these trends may be linked to region-specific factors (16). On the one hand, although Australia and New Zealand have some of the highest ultraviolet indices worldwide, long-standing sun protection campaigns have reduced cutaneous vitamin D synthesis, and seasonal or high-latitude variations further exacerbate vitamin D deficiency in women, particularly in FHFPW population (5, 31). On the other hand, female obesity rates in Oceania rank among the highest globally, especially in Pacific Island nations (32). Obesity not only increases the risk of falls and hip fractures but is also frequently accompanied by vitamin D deficiency, sarcopenia, and metabolic syndrome, all of which compromise bone health (33). These findings underscore the need for public health strategies in Oceania, including education on appropriate sun exposure and weight management, to reduce the burden of FHFPW (34).

Although the prevalence of FHFPW in the 45–49 and 50–54 age groups across the five SDI regions showed an overall downward trend over the past 32 years, short-term increases were observed in most regions (except high-SDI) during 2019–2021, likely reflecting the impact of the COVID-19 pandemic (35, 36). Reduced sunlight exposure, decreased physical activity, medication shortages, and increased use of hormone therapies may have jointly contributed to this transient surge (37–40). However, in high—and middle-SDI regions, both incidence and prevalence increased, with the rise in prevalence being more pronounced than that in incidence. This pattern suggests a relatively low mortality risk and indicates that peri-menopausal women often survive long after experiencing hip fractures. Such findings underscore the importance of optimizing surgical interventions, postoperative management, and rehabilitation to improve long-term outcomes and quality of life. The declining trend in YLDs rates further supports this interpretation, suggesting that the years of healthy life lost due to hip fractures among peri-menopausal women are gradually decreasing. Overall, despite the continuing substantial burden of disease and incidence, current health policies, treatment strategies, and rehabilitation programs appear to have had a positive impact on postoperative outcomes and late-life quality of life (41–44).

We further examined cross-country variations in the burden of FHFPW from 1990 to 2021. Differences in health policies, financial investment, medical resources, and postoperative rehabilitation quality appear to directly influence disease burden (19, 27, 45, 46). Substantial heterogeneity was observed in the 50–54 age group: while most countries experienced an increasing burden, notable declines were concentrated in Northern and Eastern Europe, Central Asia, and Mediterranean regions, often among geographically proximate nations. Such patterns may reflect shared ethnic backgrounds, dietary habits, and the exchange of health policy strategies. In Europe, for instance, policy coordination is partly facilitated by European Union membership, whereas neighboring non-EU countries also exhibit parallel trends. However, there are also country-level outliers: for example, the Russian Federation and Belarus, despite their geographic proximity, show marked differences in the annual incidence of hip fractures among perimenopausal women, suggesting that country-specific factors such as policy implementation, healthcare accessibility, registration/coding practices, or environmental conditions may also play an important role (47–49). These findings suggest that both regional collaboration and population-specific characteristics influence the FHFPW burden.

Improving the identification of potential FHFPW is essential, and governments should establish healthcare policies that include, but are not limited to, the collection of dual-energy X-ray absorptiometry (DEXA) data from perimenopausal women, the establishment of databases, the sharing of data with family or community physicians, and the ongoing follow-up of key populations. At the same time, trauma centers should be established at all levels of hospitals, especially primary health care institutions, to improve the mechanism of patient transportation and to operate on patients within 24–48 h after injury, depending on their conditions. In addition, it is necessary to strengthen the working environment, living environment, and road facilities to reduce the risk of related falls.

## 5 Limitation

This study has several limitations. First, both incidence, prevalence and YLDs estimates were derived from registry-based GBD data rather than direct case ascertainment, and the true numbers may therefore differ due to potential underreporting, misclassification, or variations in coding practices. Second, the selected age groups (45–49 and 50–54 years) may not fully capture the typical burden of hip fractures, which usually peaks after 70 years of age. Third, the analysis did not differentiate individual ICD-10 codes but relied on broader clusters, which may reduce diagnostic specificity. These factors should be considered when interpreting our findings, and future population-based prospective studies are warranted to validate these observations.

## 6 Conclusion

The global burden of disease for fall-induced hip fractures in perimenopausal women shows an overall increasing trend from 1990 to 2021. The overall burden of disease is higher in the 50–54 age group than in the 45–49 age group. It is worth noting that the number of prevalence cases is increasing in most countries and regions, and although YLDs rates and EAPCs are decreasing in most countries and regions due to good health care development, the large increases in high-income North America and Oceania should not be ignored. There is also significant heterogeneity between regions with different SDI levels, with countries with high SDI generally showing higher prevalence, incidence and YLDs.

## Data Availability

The original contributions presented in the study are included in the article/Supplementary material, further inquiries can be directed to the corresponding authors.
