# Supplementary material for: Global trends in fall-induced hip fractures among perimenopausal women 1990 to 2021: results from the global burden of disease study 2021
Source: Front Public Health. 2025 Oct 14;13:1674535. doi: 10.3389/fpubh.2025.1674535 (PMC12558873; doi:10.3389/fpubh.2025.1674535)
Supplement: Supplementary file 1 [file Table_1.docx]

Table S1. Incidence of fall-induced hip fractures in perimenopausal women in 1990 and 2021 and change from 1990 to 2021.

| **location** | **Counts** | | | **Rate** | | | |
| --- | --- | --- | --- | --- | --- | --- | --- |
|  | **1990 (95%CI)** | **2021 (95%CI)** | **Percentage change (100%, 95%UI)** | **1990 (95%CI)** | **2021 (95%CI)** | **Percentage change (100%, 95%UI)** | **EAPC (100%, 95%UI)** |
| **45-49 years** |  |  |  |  |  |  |  |
| Global | 58274.8483 (31916.262 to 99141.3877) | 113310.8825 (63120.2843 to 189456.4934) | 0.9444 (0.1486 to 1.7402) | 51.2078 (28.0458 to 87.1185) | 48.0859 (26.7865 to 80.3999) | -0.061 (-0.4453 to 0.3233) | -0.32 (-0.38 to -0.27) |
| High SDI | 23395.0386 (13112.5754 to 38947.931) | 36923.7067 (20921.268 to 62197.2236) | 0.5783 (-0.0544 to 1.211) | 92.5926 (51.8968 to 154.1476) | 102.8342 (58.2667 to 173.2221) | 0.1106 (-0.3346 to 0.5558) | 0.46 (0.37-0.55) |
| High-middle SDI | 14413.893 (7813.5464 to 24598.6986) | 26105.4356 (14487.209 to 43584.081) | 0.8111 (0.0663 to 1.5559) | 58.7905 (31.8694 to 100.3316) | 54.1292 (30.039 to 90.3709) | -0.0793 (-0.4579 to 0.2993) | -0.48 (-0.59 to -0.38) |
| Middle SDI | 11137.6071 (5999.1795 to 19436.5731) | 29716.2135 (16267.4971 to 50080.01) | 1.6681 (0.5393 to 2.7969) | 32.8611 (17.7003 to 57.3468) | 36.6364 (20.0558 to 61.7425) | 0.1149 (-0.3568 to 0.5866) | 0.19 (-0.04-0.43) |
| Low-middle SDI | 7352.5109 (3928.9062 to 12827.0866) | 15689.5669 (8651.9489 to 26215.4501) | 1.1339 (0.2365 to 2.0313) | 33.8745 (18.1013 to 59.097) | 31.7365 (17.5009 to 53.0279) | -0.0631 (-0.4571 to 0.3309) | -0.28 (-0.32 to -0.25) |
| Low SDI | 1915.0065 (1036.0142 to 3297.9518) | 4794.233 (2654.2733 to 8147.8195) | 1.5035 (0.4525 to 2.5545) | 23.0677 (12.4796 to 39.7264) | 23.0853 (12.7809 to 39.2336) | 0.0008 (-0.4193 to 0.4209) | -0.09 (-0.14 to -0.05) |
| Andean Latin America | 124.0481 (66.0593 to 210.6573) | 432.0045 (237.143 to 736.683) | 2.4826 (1.0239 to 3.9413) | 17.4512 (9.2933 to 29.6355) | 23.0933 (12.6767 to 39.3802) | 0.3233 (-0.231 to 0.8776) | 0.84 (0.73-0.95) |
| Australasia | 444.9256 (237.1461 to 789.4388) | 843.0644 (455.597 to 1459.3249) | 0.8948 (0.0634 to 1.7262) | 78.5871 (41.8871 to 139.4384) | 83.6549 (45.2076 to 144.8047) | 0.0645 (-0.4026 to 0.5316) | 0.34 (0.22-0.45) |
| Caribbean | 134.9325 (70.155 to 243.9304) | 302.8783 (161.3047 to 535.2439) | 1.2447 (0.2231 to 2.2663) | 17.0859 (8.8834 to 30.8879) | 20.8618 (11.1104 to 36.8668) | 0.221 (-0.3347 to 0.7767) | 0.71 (0.48-0.94) |
| Central Asia | 371.5057 (206.8508 to 618.7172) | 744.259 (418.4994 to 1227.7234) | 1.0034 (0.2098 to 1.797) | 31.7495 (17.6778 to 52.8765) | 27.0963 (15.2363 to 44.6978) | -0.1466 (-0.4847 to 0.1915) | -0.46 (-0.55 to -0.37) |
| Central Europe | 2406.6432 (1364.2371 to 3980.7797) | 2474.1669 (1376.4614 to 4091.2028) | 0.0281 (-0.377 to 0.4332) | 70.5959 (40.0182 to 116.7713) | 57.7484 (32.1274 to 95.491) | -0.182 (-0.5043 to 0.1403) | -0.84 (-0.98 to -0.7) |
| Central Latin America | 1244.055 (669.5958 to 2164.8987) | 2419.1965 (1309.4823 to 4175.335) | 0.9446 (0.1074 to 1.7818) | 41.5516 (22.3646 to 72.3079) | 30.5041 (16.5115 to 52.6475) | -0.2659 (-0.582 to 0.0502) | -0.54 (-0.79 to -0.3) |
| Central Sub-Saharan Africa | 104.7422 (54.4986 to 181.7181) | 326.4639 (167.2142 to 556.1763) | 2.1168 (0.764 to 3.4696) | 11.6556 (6.0646 to 20.2214) | 13.1417 (6.7312 to 22.3887) | 0.1275 (-0.3619 to 0.6169) | 0.36 (0.3-0.42) |
| East Asia | 9728.0105 (5176.5676 to 17029.5107) | 26200.319 (14399.5639 to 44362.3621) | 1.6933 (0.5452 to 2.8414) | 38.2079 (20.3316 to 66.8855) | 46.6792 (25.6546 to 79.0371) | 0.2217 (-0.2991 to 0.7425) | 0.4 (-0.03-0.83) |
| Eastern Europe | 4446.7798 (2422.3827 to 7486.7816) | 4971.0067 (2768.4102 to 8121.1688) | 0.1179 (-0.3291 to 0.5649) | 78.8222 (42.9384 to 132.7083) | 65.68 (36.5779 to 107.3019) | -0.1667 (-0.4999 to 0.1665) | -0.95 (-1.34 to -0.57) |
| Eastern Sub-Saharan Africa | 361.0617 (185.6751 to 632.9793) | 899.7151 (470.2738 to 1558.8224) | 1.4919 (0.3911 to 2.5927) | 12.5342 (6.4457 to 21.9738) | 11.9391 (6.2405 to 20.6853) | -0.0475 (-0.4683 to 0.3733) | -0.27 (-0.32 to -0.21) |
| High-income Asia Pacific | 4147.2647 (2282.9527 to 7062.91) | 4678.9021 (2596.1387 to 8079.1042) | 0.1282 (-0.3449 to 0.6013) | 71.4854 (39.3507 to 121.7417) | 65.2303 (36.1937 to 112.6338) | -0.0875 (-0.4701 to 0.2951) | -0.27 (-0.49 to -0.06) |
| High-income North America | 7280.7024 (3966.6869 to 12647.9297) | 15699.1922 (8834.1236 to 26536.3887) | 1.1563 (0.2536 to 2.059) | 91.9224 (50.0813 to 159.6863) | 140.5338 (79.0801 to 237.5447) | 0.5288 (-0.1112 to 1.1688) | 1.65 (1.53-1.78) |
| North Africa and Middle East | 1062.1732 (575.4982 to 1791.9998) | 3947.011 (2167.0311 to 6533.5368) | 2.716 (1.2065 to 4.2255) | 18.3826 (9.9599 to 31.0134) | 23.3741 (12.8331 to 38.6914) | 0.2715 (-0.245 to 0.788) | 0.64 (0.54-0.75) |
| Oceania | 46.949 (25.7594 to 79.6387) | 191.4657 (107.6582 to 315.0164) | 3.0782 (1.4366 to 4.7198) | 40.8769 (22.4278 to 69.3387) | 59.2177 (33.2972 to 97.4302) | 0.4487 (-0.1345 to 1.0319) | 1.12 (1.07-1.16) |
| South Asia | 8909.1506 (4796.3615 to 15630.806) | 21087.0623 (11518.4303 to 35381.0674) | 1.3669 (0.3639 to 2.3699) | 42.9836 (23.1408 to 75.4132) | 42.7998 (23.3786 to 71.8118) | -0.0043 (-0.4263 to 0.4177) | -0.07 (-0.12 to -0.02) |
| Southeast Asia | 2131.9775 (1140.9323 to 3707.9711) | 4920.6799 (2677.7658 to 8291.9337) | 1.308 (0.3313 to 2.2847) | 23.1418 (12.3844 to 40.2486) | 22.0768 (12.0139 to 37.202) | -0.046 (-0.4497 to 0.3577) | -0.35 (-0.42 to -0.28) |
| Southern Latin America | 587.6763 (312.2146 to 1031.6017) | 1216.7858 (673.5415 to 2141.6336) | 1.0705 (0.1627 to 1.9783) | 45.7795 (24.3213 to 80.361) | 55.6689 (30.8151 to 97.9814) | 0.216 (-0.3172 to 0.7492) | 0.68 (0.38-0.99) |
| Southern Sub-Saharan Africa | 93.7445 (47.2342 to 172.6973) | 173.3183 (87.486 to 318.266) | 0.8488 (-0.0416 to 1.7392) | 9.8498 (4.9629 to 18.1455) | 8.2476 (4.1631 to 15.1451) | -0.1627 (-0.566 to 0.2406) | -0.5 (-0.58 to -0.42) |
| Tropical Latin America | 1340.8163 (718.5804 to 2340.8917) | 3298.7807 (1834.6111 to 5515.7154) | 1.4603 (0.4273 to 2.4933) | 42.0448 (22.533 to 73.4048) | 43.8297 (24.3758 to 73.2852) | 0.0425 (-0.3952 to 0.4802) | -0.09 (-0.41-0.22) |
| Western Europe | 12909.663 (7233.3346 to 21266.0157) | 17278.6015 (9583.6633 to 28632.9525) | 0.3384 (-0.1902 to 0.867) | 113.8475 (63.7892 to 187.5403) | 115.8282 (64.2447 to 191.9427) | 0.0174 (-0.3844 to 0.4192) | 0.03 (-0.11-0.17) |
| Western Sub-Saharan Africa | 398.0267 (207.2541 to 702.9056) | 1206.0089 (625.5653 to 2067.1163) | 2.03 (0.6958 to 3.3642) | 13.4431 (6.9999 to 23.7402) | 13.7192 (7.1163 to 23.515) | 0.0205 (-0.4289 to 0.4699) | 0.08 (0.01-0.15) |
| **50-54 years** |  |  |  |  |  |  |  |
| Global | 90702.0704 (50815.4513 to 150723.0229) | 194780.8851 (110009.124 to 316287.232) | 1.1475 (0.3104 to 1.9846) | 86.4514 (48.434 to 143.6596) | 87.3682 (49.3442 to 141.8694) | 0.0106 (-0.3833 to 0.4045) | 0.05 (-0.01-0.1) |
| High SDI | 37485.8627 (21632.9835 to 60258.311) | 76549.7025 (44892.9045 to 120000.9572) | 1.0421 (0.3009 to 1.7833) | 160.4789 (92.6119 to 257.9689) | 207.9655 (121.9623 to 326.0112) | 0.2959 (-0.1745 to 0.7663) | 1.03 (0.96-1.1) |
| High-middle SDI | 24030.0501 (13398.6614 to 40754.5547) | 40146.9328 (22329.2369 to 66468.1752) | 0.6707 (-0.0038 to 1.3452) | 89.8614 (50.1049 to 152.4034) | 82.8246 (46.066 to 137.1263) | -0.0783 (-0.4504 to 0.2938) | -0.38 (-0.45 to -0.31) |
| Middle SDI | 15631.6194 (8327.7021 to 27093.8779) | 47696.6141 (25753.2074 to 80012.424) | 2.0513 (0.7639 to 3.3387) | 52.6452 (28.0466 to 91.2485) | 60.4386 (32.6331 to 101.3875) | 0.148 (-0.3364 to 0.6324) | 0.35 (0.11-0.59) |
| Low-middle SDI | 10686.0539 (5714.4237 to 18591.4401) | 23480.0272 (12814.695 to 39437.9312) | 1.1973 (0.2698 to 2.1248) | 58.9205 (31.508 to 102.509) | 55.5201 (30.3012 to 93.2536) | -0.0577 (-0.4554 to 0.34) | -0.24 (-0.32 to -0.17) |
| Low SDI | 2772.7412 (1539.6207 to 4653.8333) | 6780.2868 (3797.7749 to 10929.4707) | 1.4453 (0.4854 to 2.4052) | 40.3248 (22.3912 to 67.6821) | 41.6573 (23.3331 to 67.1494) | 0.033 (-0.3725 to 0.4385) | 0.11 (0.07-0.16) |
| Andean Latin America | 156.0299 (84.8029 to 262.132) | 569.1332 (309.5995 to 945.4451) | 2.6476 (1.1647 to 4.1305) | 26.9366 (14.6402 to 45.2538) | 35.3373 (19.2229 to 58.7024) | 0.3119 (-0.2214 to 0.8452) | 0.82 (0.72-0.92) |
| Australasia | 602.4603 (312.1916 to 1043.4688) | 1445.9817 (810.1964 to 2338.1156) | 1.4001 (0.4148 to 2.3854) | 127.6293 (66.1368 to 221.0555) | 144.6711 (81.0605 to 233.9296) | 0.1335 (-0.3319 to 0.5989) | 0.59 (0.49-0.69) |
| Caribbean | 212.012 (113.8736 to 362.9765) | 582.5291 (320.0523 to 974.0052) | 1.7476 (0.6086 to 2.8866) | 31.9879 (17.181 to 54.7651) | 41.5525 (22.8297 to 69.4769) | 0.299 (-0.2395 to 0.8375) | 0.75 (0.58-0.93) |
| Central Asia | 658.324 (375.1156 to 1083.6651) | 904.592 (501.3377 to 1468.5315) | 0.3741 (-0.1577 to 0.9059) | 42.8407 (24.4108 to 70.5199) | 36.0604 (19.9852 to 58.541) | -0.1583 (-0.4841 to 0.1675) | -0.5 (-0.58 to -0.42) |
| Central Europe | 3701.64 (2169.3474 to 5922.0563) | 3390.6717 (1947.592 to 5507.4873) | -0.084 (-0.425 to 0.257) | 102.5059 (60.0736 to 163.9937) | 86.6031 (49.7446 to 140.67) | -0.1551 (-0.4697 to 0.1595) | -0.84 (-0.98 to -0.71) |
| Central Latin America | 1749.9978 (945.4801 to 3007.3531) | 3563.1443 (1967.8908 to 5932.6262) | 1.0361 (0.1944 to 1.8778) | 71.0491 (38.3861 to 122.0972) | 49.7063 (27.4523 to 82.7609) | -0.3004 (-0.5896 to -0.0112) | -0.89 (-1.08 to -0.7) |
| Central Sub-Saharan Africa | 199.6845 (115.9942 to 324.2413) | 560.6637 (334.5483 to 901.6787) | 1.8077 (0.7671 to 2.8483) | 25.1379 (14.6023 to 40.8181) | 27.8446 (16.6149 to 44.7806) | 0.1077 (-0.3028 to 0.5182) | 0.38 (0.31-0.46) |
| East Asia | 13729.9246 (7205.8189 to 23978.1675) | 43799.3501 (23724.9994 to 73642.0719) | 2.1901 (0.8305 to 3.5497) | 58.3848 (30.6418 to 101.9642) | 70.9704 (38.4428 to 119.3261) | 0.2156 (-0.3025 to 0.7337) | 0.69 (0.36-1.02) |
| Eastern Europe | 8299.2209 (4510.6634 to 14380.6115) | 5714.1149 (3186.2575 to 9567.8117) | -0.3115 (-0.598 to -0.025) | 97.6253 (53.0598 to 169.1618) | 82.5972 (46.0572 to 138.3022) | -0.1539 (-0.506 to 0.1982) | -1.07 (-1.52 to -0.62) |
| Eastern Sub-Saharan Africa | 605.8408 (337.5247 to 1008.5162) | 1375.1905 (792.8889 to 2210.1341) | 1.2699 (0.3939 to 2.1459) | 25.5826 (14.2525 to 42.5863) | 24.2437 (13.9781 to 38.9631) | -0.0523 (-0.418 to 0.3134) | -0.21 (-0.29 to -0.14) |
| High-income Asia Pacific | 5782.088 (3179.7009 to 9664.4583) | 7491.2447 (4076.4149 to 12393.9978) | 0.2956 (-0.226 to 0.8172) | 110.1975 (60.6001 to 184.1894) | 105.7352 (57.5366 to 174.9351) | -0.0405 (-0.4268 to 0.3458) | -0.11 (-0.29-0.06) |
| High-income North America | 10361.7604 (5575.7021 to 17663.3249) | 36422.3568 (21100.5708 to 58108.5231) | 2.5151 (1.1279 to 3.9023) | 158.2921 (85.1775 to 269.8348) | 307.2909 (178.0229 to 490.2544) | 0.9413 (0.1752 to 1.7074) | 2.57 (2.36-2.78) |
| North Africa and Middle East | 1223.7075 (653.0392 to 2066.8377) | 4354.8272 (2436.8754 to 7225.609) | 2.5587 (1.1107 to 4.0067) | 24.5914 (13.1233 to 41.5348) | 31.5571 (17.6587 to 52.3602) | 0.2833 (-0.2388 to 0.8054) | 0.68 (0.56-0.79) |
| Oceania | 55.6415 (30.5917 to 92.5926) | 219.8844 (126.3951 to 354.3253) | 2.9518 (1.4176 to 4.486) | 60.3903 (33.2026 to 100.4951) | 85.6498 (49.2336 to 138.0174) | 0.4183 (-0.1323 to 0.9689) | 0.99 (0.94-1.05) |
| South Asia | 12867.7238 (6769.5214 to 22481.1093) | 32212.1988 (17378.2217 to 53560.8906) | 1.5033 (0.4438 to 2.5628) | 76.2431 (40.1104 to 133.2037) | 76.7938 (41.4297 to 127.6891) | 0.0072 (-0.4191 to 0.4335) | 0.01 (-0.11-0.13) |
| Southeast Asia | 3332.8289 (1839.492 to 5609.0309) | 8084.6463 (4613.4826 to 13189.8173) | 1.4258 (0.4662 to 2.3854) | 40.8026 (22.5202 to 68.6694) | 39.5599 (22.5747 to 64.5405) | -0.0305 (-0.414 to 0.353) | -0.37 (-0.45 to -0.28) |
| Southern Latin America | 827.1542 (450.0148 to 1381.0349) | 1725.1731 (928.0411 to 2874.608) | 1.0857 (0.2377 to 1.9337) | 72.8774 (39.6491 to 121.6776) | 88.0115 (47.345 to 146.6511) | 0.2077 (-0.2833 to 0.6987) | 0.69 (0.44-0.93) |
| Southern Sub-Saharan Africa | 126.7797 (65.0539 to 220.7185) | 240.5591 (126.5746 to 414.9588) | 0.8975 (0.0669 to 1.7281) | 15.3678 (7.8856 to 26.7547) | 13.5598 (7.1348 to 23.3904) | -0.1176 (-0.5039 to 0.2687) | -0.34 (-0.4 to -0.28) |
| Tropical Latin America | 1713.3097 (917.5393 to 2990.5266) | 4701.6839 (2561.4088 to 7875.9513) | 1.7442 (0.5851 to 2.9033) | 63.4516 (33.9806 to 110.7526) | 68.5019 (37.3188 to 114.7499) | 0.0796 (-0.3764 to 0.5356) | 0.08 (-0.19-0.35) |
| Western Europe | 23843.2186 (14252.7834 to 37525.3551) | 35388.7368 (21154.8176 to 55132.2924) | 0.4842 (-0.0342 to 1.0026) | 207.6876 (124.1496 to 326.8665) | 222.6282 (133.0836 to 346.8336) | 0.0719 (-0.3025 to 0.4463) | 0.27 (0.14-0.39) |
| Western Sub-Saharan Africa | 652.7233 (365.9073 to 1076.7173) | 2034.2027 (1157.9234 to 3262.2375) | 2.1165 (0.9224 to 3.3106) | 27.6024 (15.4735 to 45.5323) | 28.4552 (16.1975 to 45.6334) | 0.0309 (-0.3641 to 0.4259) | 0.13 (0.07-0.19) |
