# Supplementary material for: Global trends in fall-induced hip fractures among perimenopausal women 1990 to 2021: results from the global burden of disease study 2021
Source: Front Public Health. 2025 Oct 14;13:1674535. doi: 10.3389/fpubh.2025.1674535 (PMC12558873; doi:10.3389/fpubh.2025.1674535)
Supplement: Supplementary file 3 [file Table_3.docx]

Table S2. YLDs of fall-induced hip fractures in perimenopausal women in 1990 and 2021 and change from 1990 to 2021.

| **location** | **Counts** | | | **Rate** | | | |
| --- | --- | --- | --- | --- | --- | --- | --- |
|  | **1990 (95%CI)** | **2021 (95%CI)** | **Percentage change (100%, 95%UI)** | **1990 (95%CI)** | **2021 (95%CI)** | **Percentage change (100%, 95%UI)** | **EAPC (100%, 95%UI)** |
| **45-49 years** |  |  |  |  |  |  |  |
| Global | 14932.0833 (10115.9098 to 20295.6047) | 19702.3865 (13187.6819 to 28070.183) | 0.3195 (-0.023 to 0.662) | 13.1213 (8.8891 to 17.8343) | 8.3611 (5.5965 to 11.9122) | -0.3628 (-0.5282 to -0.1974) | -1.68 (-1.77 to -1.6) |
| High SDI | 4008.3371 (2585.4912 to 5773.7139) | 4641.6188 (2910.6668 to 7175.2309) | 0.158 (-0.201 to 0.517) | 15.8641 (10.2328 to 22.8511) | 12.9271 (8.1063 to 19.9833) | -0.1851 (-0.4377 to 0.0675) | -0.44 (-0.53 to -0.34) |
| High-middle SDI | 3411.2354 (2254.4318 to 4700.1559) | 3578.0443 (2316.5745 to 5378.779) | 0.0489 (-0.2498 to 0.3476) | 13.9135 (9.1952 to 19.1707) | 7.419 (4.8034 to 11.1528) | -0.4668 (-0.6187 to -0.3149) | -2.6 (-2.85 to -2.36) |
| Middle SDI | 3741.2022 (2552.5998 to 5109.2606) | 5382.1624 (3595.9776 to 7541.1845) | 0.4386 (0.0708 to 0.8064) | 11.0383 (7.5313 to 15.0747) | 6.6355 (4.4334 to 9.2974) | -0.3989 (-0.5526 to -0.2452) | -2.03 (-2.24 to -1.82) |
| Low-middle SDI | 2964.2847 (2002.9421 to 4124.1648) | 4547.7806 (3078.5286 to 6228.3339) | 0.5342 (0.1444 to 0.924) | 13.6571 (9.228 to 19.0009) | 9.1991 (6.2272 to 12.5985) | -0.3264 (-0.4975 to -0.1553) | -1.42 (-1.48 to -1.35) |
| Low SDI | 793.2962 (518.8199 to 1122.179) | 1539.2273 (1021.2446 to 2139.4878) | 0.9403 (0.4197 to 1.4609) | 9.5559 (6.2496 to 13.5175) | 7.4117 (4.9175 to 10.3021) | -0.2244 (-0.4325 to -0.0163) | -0.91 (-1 to -0.82) |
| Andean Latin America | 46.5161 (27.9797 to 68.1447) | 89.2698 (56.0651 to 135.1749) | 0.9191 (0.3134 to 1.5248) | 6.5439 (3.9362 to 9.5867) | 4.772 (2.997 to 7.2259) | -0.2708 (-0.501 to -0.0406) | -1.09 (-1.2 to -0.97) |
| Australasia | 74.1945 (44.6928 to 112.0909) | 132.2471 (77.0141 to 201.2138) | 0.7824 (0.1883 to 1.3765) | 13.105 (7.8941 to 19.7986) | 13.1225 (7.6419 to 19.9659) | 0.0013 (-0.3325 to 0.3351) | 0.15 (0.01-0.28) |
| Caribbean | 29.534 (17.6145 to 43.7975) | 52.2282 (33.3902 to 76.1997) | 0.7684 (0.2237 to 1.3131) | 3.7398 (2.2304 to 5.5459) | 3.5974 (2.2999 to 5.2485) | -0.0381 (-0.3344 to 0.2582) | -0.12 (-0.22 to -0.01) |
| Central Asia | 116.5847 (78.623 to 162.475) | 181.7057 (115.8843 to 264.832) | 0.5586 (0.125 to 0.9922) | 9.9635 (6.7192 to 13.8854) | 6.6154 (4.219 to 9.6418) | -0.336 (-0.5207 to -0.1513) | -1.35 (-1.45 to -1.26) |
| Central Europe | 536.6166 (349.4332 to 753.9874) | 298.1094 (187.0128 to 451.6501) | -0.4445 (-0.6096 to -0.2794) | 15.741 (10.2502 to 22.1173) | 6.958 (4.365 to 10.5418) | -0.558 (-0.6893 to -0.4267) | -2.8 (-2.87 to -2.73) |
| Central Latin America | 383.0857 (251.5515 to 532.1502) | 490.3473 (324.3038 to 683.2589) | 0.28 (-0.0581 to 0.6181) | 12.7951 (8.4019 to 17.7739) | 6.1829 (4.0892 to 8.6153) | -0.5168 (-0.6445 to -0.3891) | -1.71 (-1.96 to -1.45) |
| Central Sub-Saharan Africa | 38.8986 (22.9484 to 63.8561) | 101.8907 (58.398 to 161.802) | 1.6194 (0.6428 to 2.596) | 4.3286 (2.5537 to 7.1058) | 4.1016 (2.3508 to 6.5133) | -0.0524 (-0.4057 to 0.3009) | -0.09 (-0.19-0.02) |
| East Asia | 2867.9369 (1929.1047 to 3967.0135) | 3196.1463 (2026.9027 to 4892.4284) | 0.1144 (-0.2108 to 0.4396) | 11.2642 (7.5768 to 15.5809) | 5.6943 (3.6112 to 8.7165) | -0.4945 (-0.642 to -0.347) | -3.14 (-3.84 to -2.44) |
| Eastern Europe | 963.6923 (643.2375 to 1365.7066) | 730.8317 (478.906 to 1067.5123) | -0.2416 (-0.4545 to -0.0287) | 17.0821 (11.4018 to 24.2081) | 9.6562 (6.3276 to 14.1046) | -0.4347 (-0.5934 to -0.276) | -2.37 (-2.95 to -1.79) |
| Eastern Sub-Saharan Africa | 136.6206 (89.7402 to 196.489) | 290.6038 (189.8111 to 413.4214) | 1.1271 (0.532 to 1.7222) | 4.7428 (3.1153 to 6.8211) | 3.8563 (2.5188 to 5.486) | -0.1869 (-0.4144 to 0.0406) | -0.54 (-0.63 to -0.45) |
| High-income Asia Pacific | 933.0413 (606.2073 to 1321.8303) | 742.8267 (469.07 to 1148.2553) | -0.2039 (-0.4463 to 0.0385) | 16.0826 (10.4491 to 22.7841) | 10.356 (6.5395 to 16.0083) | -0.3561 (-0.5521 to -0.1601) | -1.27 (-1.36 to -1.17) |
| High-income North America | 1051.4595 (667.9737 to 1598.8595) | 1699.4407 (1047.2851 to 2675.8805) | 0.6163 (0.0784 to 1.1542) | 13.2752 (8.4335 to 20.1864) | 15.2128 (9.3749 to 23.9536) | 0.146 (-0.2354 to 0.5274) | 0.63 (0.5-0.77) |
| North Africa and Middle East | 463.9931 (304.2081 to 661.5491) | 877.7383 (600.0229 to 1211.4887) | 0.8917 (0.3906 to 1.3928) | 8.0301 (5.2648 to 11.4492) | 5.1979 (3.5533 to 7.1744) | -0.3527 (-0.5242 to -0.1812) | -1.6 (-1.74 to -1.47) |
| Oceania | 15.8065 (9.5087 to 23.5764) | 58.4385 (34.8279 to 86.484) | 2.6971 (1.5141 to 3.8801) | 13.7622 (8.2789 to 20.5272) | 18.0742 (10.7718 to 26.7483) | 0.3133 (-0.107 to 0.7336) | 0.88 (0.82-0.94) |
| South Asia | 3556.1152 (2377.4548 to 4888.8549) | 5821.0371 (3889.8795 to 8055.9474) | 0.6369 (0.217 to 1.0568) | 17.157 (11.4704 to 23.587) | 11.8148 (7.8952 to 16.3509) | -0.3114 (-0.488 to -0.1348) | -1.37 (-1.44 to -1.29) |
| Southeast Asia | 716.5165 (480.3448 to 996.1203) | 1157.4888 (763.8257 to 1625.344) | 0.6154 (0.1887 to 1.0421) | 7.7775 (5.214 to 10.8125) | 5.1931 (3.4269 to 7.2921) | -0.3323 (-0.5087 to -0.1559) | -1.56 (-1.64 to -1.47) |
| Southern Latin America | 270.0476 (175.7739 to 389.2718) | 305.3895 (193.3909 to 448.6774) | 0.1309 (-0.201 to 0.4628) | 21.0365 (13.6927 to 30.324) | 13.9718 (8.8478 to 20.5273) | -0.3358 (-0.5307 to -0.1409) | -1.33 (-1.51 to -1.14) |
| Southern Sub-Saharan Africa | 37.2301 (24.0512 to 52.8032) | 46.8696 (29.6446 to 69.582) | 0.2589 (-0.1104 to 0.6282) | 3.9118 (2.5271 to 5.5481) | 2.2303 (1.4107 to 3.3111) | -0.4299 (-0.5972 to -0.2626) | -1.84 (-2.07 to -1.62) |
| Tropical Latin America | 472.8003 (310.8181 to 667.3065) | 705.8634 (465.5144 to 1005.7121) | 0.4929 (0.0837 to 0.9021) | 14.8259 (9.7465 to 20.9251) | 9.3785 (6.1851 to 13.3625) | -0.3674 (-0.5408 to -0.194) | -1.61 (-1.88 to -1.34) |
| Western Europe | 2078.2794 (1305.6436 to 3056.7744) | 2372.8329 (1475.6384 to 3571.0735) | 0.1417 (-0.2138 to 0.4972) | 18.3279 (11.5142 to 26.957) | 15.9064 (9.892 to 23.9389) | -0.1321 (-0.4023 to 0.1381) | -0.2 (-0.33 to -0.07) |
| Western Sub-Saharan Africa | 143.1138 (91.5701 to 201.2037) | 351.081 (230.6656 to 497.7132) | 1.4532 (0.7776 to 2.1288) | 4.8336 (3.0927 to 6.7955) | 3.9938 (2.624 to 5.6619) | -0.1737 (-0.4013 to 0.0539) | -0.51 (-0.58 to -0.44) |
| **50-54 years** |  |  |  |  |  |  |  |
| Global | 23488.0953 (15938.6318 to 33394.6769) | 33335.8949 (22259.1306 to 47875.1483) | 0.4193 (0.0323 to 0.8063) | 22.3874 (15.1917 to 31.8297) | 14.9527 (9.9842 to 21.4742) | -0.3321 (-0.5142 to -0.15) | -1.4 (-1.47 to -1.34) |
| High SDI | 6728.9372 (4508.5042 to 9812.6012) | 9970.559 (6145.5333 to 14934.9555) | 0.4817 (0.0347 to 0.9287) | 28.8069 (19.3011 to 42.0083) | 27.0874 (16.6958 to 40.5744) | -0.0597 (-0.3434 to 0.224) | 0.1 (-0.03-0.22) |
| High-middle SDI | 6106.4418 (4134.874 to 8751.3959) | 5788.2226 (3779.1833 to 8528.9362) | -0.0521 (-0.3219 to 0.2177) | 22.8353 (15.4625 to 32.7262) | 11.9413 (7.7966 to 17.5955) | -0.4771 (-0.6259 to -0.3283) | -2.49 (-2.68 to -2.3) |
| Middle SDI | 5434.2257 (3598.4432 to 7675.1752) | 8817.7912 (5886.9674 to 12553.5346) | 0.6226 (0.1817 to 1.0635) | 18.3017 (12.1191 to 25.849) | 11.1734 (7.4597 to 15.9072) | -0.3895 (-0.5554 to -0.2236) | -1.88 (-2.07 to -1.69) |
| Low-middle SDI | 4105.7658 (2720.4234 to 5894.533) | 6614.2305 (4518.7143 to 9230.5604) | 0.611 (0.179 to 1.043) | 22.6383 (14.9998 to 32.5011) | 15.6398 (10.6848 to 21.8263) | -0.3091 (-0.4944 to -0.1238) | -1.31 (-1.36 to -1.26) |
| Low SDI | 1089.9578 (722.9906 to 1568.7678) | 2123.5993 (1450.8444 to 2937.7923) | 0.9483 (0.4288 to 1.4678) | 15.8516 (10.5147 to 22.8151) | 13.0471 (8.9138 to 18.0494) | -0.1769 (-0.3964 to 0.0426) | -0.67 (-0.75 to -0.59) |
| Andean Latin America | 61.9084 (38.8757 to 88.9946) | 128.3238 (78.9697 to 188.5996) | 1.0728 (0.4504 to 1.6952) | 10.6877 (6.7114 to 15.3638) | 7.9676 (4.9032 to 11.7101) | -0.2545 (-0.4783 to -0.0307) | -1.03 (-1.15 to -0.92) |
| Australasia | 101.035 (62.1468 to 153.9727) | 226.831 (136.3753 to 340.3536) | 1.2451 (0.5128 to 1.9774) | 21.4039 (13.1656 to 32.6186) | 22.6945 (13.6444 to 34.0525) | 0.0603 (-0.2855 to 0.4061) | 0.35 (0.23-0.47) |
| Caribbean | 47.9265 (30.0748 to 70.3412) | 96.7042 (60.7493 to 140.3094) | 1.0178 (0.4125 to 1.6231) | 7.231 (4.5376 to 10.6129) | 6.898 (4.3333 to 10.0084) | -0.0461 (-0.3323 to 0.2401) | -0.21 (-0.31 to -0.11) |
| Central Asia | 227.0743 (148.8485 to 327.2117) | 244.2083 (162.1874 to 341.4973) | 0.0755 (-0.2195 to 0.3705) | 14.7769 (9.6864 to 21.2934) | 9.735 (6.4654 to 13.6133) | -0.3412 (-0.5219 to -0.1605) | -1.38 (-1.49 to -1.27) |
| Central Europe | 902.0296 (608.7482 to 1281.9835) | 441.1826 (277.962 to 662.4694) | -0.5109 (-0.6541 to -0.3677) | 24.979 (16.8575 to 35.5007) | 11.2685 (7.0996 to 16.9205) | -0.5489 (-0.6809 to -0.4169) | -2.82 (-2.91 to -2.73) |
| Central Latin America | 549.9348 (367.3199 to 764.8858) | 762.5144 (500.2252 to 1081.5529) | 0.3866 (0.015 to 0.7582) | 22.3271 (14.913 to 31.054) | 10.6372 (6.9782 to 15.0878) | -0.5236 (-0.6513 to -0.3959) | -1.89 (-2.12 to -1.66) |
| Central Sub-Saharan Africa | 66.0682 (38.9723 to 100.288) | 160.7378 (95.4694 to 243.9201) | 1.4329 (0.6203 to 2.2455) | 8.3172 (4.9062 to 12.6251) | 7.9828 (4.7414 to 12.114) | -0.0402 (-0.3608 to 0.2804) | -0.04 (-0.11-0.03) |
| East Asia | 4433.815 (2876.6444 to 6357.7202) | 5792.3251 (3728.4868 to 8654.555) | 0.3064 (-0.0793 to 0.6921) | 18.8542 (12.2326 to 27.0354) | 9.3856 (6.0415 to 14.0234) | -0.5022 (-0.6492 to -0.3552) | -2.94 (-3.51 to -2.37) |
| Eastern Europe | 2113.6498 (1458.6075 to 3041.9436) | 953.8494 (628.0015 to 1367.0735) | -0.5487 (-0.6728 to -0.4246) | 24.8633 (17.1579 to 35.7829) | 13.7878 (9.0777 to 19.761) | -0.4455 (-0.598 to -0.293) | -2.52 (-3.12 to -1.91) |
| Eastern Sub-Saharan Africa | 203.2669 (134.1223 to 296.6026) | 405.3693 (265.8059 to 574.7545) | 0.9943 (0.4324 to 1.5562) | 8.5833 (5.6635 to 12.5245) | 7.1464 (4.686 to 10.1325) | -0.1674 (-0.402 to 0.0672) | -0.47 (-0.56 to -0.38) |
| High-income Asia Pacific | 1389.7645 (936.5926 to 1982.1892) | 1239.5019 (773.2153 to 1844.0394) | -0.1081 (-0.3687 to 0.1525) | 26.4867 (17.85 to 37.7774) | 17.495 (10.9135 to 26.0277) | -0.3395 (-0.5325 to -0.1465) | -1.13 (-1.25 to -1.01) |
| High-income North America | 1593.9789 (1006.4706 to 2391.5413) | 4313.8411 (2633.0555 to 6480.6825) | 1.7063 (0.8466 to 2.566) | 24.3505 (15.3754 to 36.5345) | 36.3953 (22.2148 to 54.6767) | 0.4946 (0.0198 to 0.9694) | 1.66 (1.46-1.86) |
| North Africa and Middle East | 547.9008 (369.1428 to 741.8304) | 1015.0836 (694.5606 to 1418.0751) | 0.8527 (0.387 to 1.3184) | 11.0105 (7.4182 to 14.9077) | 7.3558 (5.0331 to 10.276) | -0.3319 (-0.4998 to -0.164) | -1.51 (-1.65 to -1.37) |
| Oceania | 20.8775 (12.0713 to 31.1237) | 76.9231 (50.1079 to 111.9211) | 2.6845 (1.5416 to 3.8274) | 22.6594 (13.1015 to 33.78) | 29.9632 (19.5181 to 43.5957) | 0.3223 (-0.0879 to 0.7325) | 0.86 (0.8-0.92) |
| South Asia | 4881.1378 (3146.902 to 7145.1015) | 8559.4054 (5837.6934 to 12018.9923) | 0.7536 (0.2651 to 1.2421) | 28.9214 (18.6458 to 42.3357) | 20.4056 (13.9171 to 28.6533) | -0.2944 (-0.4909 to -0.0979) | -1.25 (-1.3 to -1.21) |
| Southeast Asia | 1115.4306 (739.5687 to 1570.5324) | 1874.5816 (1241.0342 to 2627.1628) | 0.6806 (0.2306 to 1.1306) | 13.6558 (9.0543 to 19.2275) | 9.1727 (6.0726 to 12.8553) | -0.3283 (-0.5082 to -0.1484) | -1.59 (-1.69 to -1.49) |
| Southern Latin America | 375.0103 (247.4694 to 560.1633) | 441.8319 (284.2322 to 650.3118) | 0.1782 (-0.1751 to 0.5315) | 33.0407 (21.8036 to 49.3538) | 22.5405 (14.5004 to 33.1763) | -0.3178 (-0.5224 to -0.1132) | -1.25 (-1.44 to -1.05) |
| Southern Sub-Saharan Africa | 49.3741 (32.8858 to 70.2704) | 64.4128 (41.6699 to 95.3023) | 0.3046 (-0.0699 to 0.6791) | 5.985 (3.9863 to 8.5179) | 3.6308 (2.3488 to 5.372) | -0.3934 (-0.5676 to -0.2192) | -1.61 (-1.81 to -1.41) |
| Tropical Latin America | 619.8451 (402.7006 to 881.2748) | 1071.8148 (695.6203 to 1549.0019) | 0.7292 (0.24 to 1.2184) | 22.9557 (14.9138 to 32.6376) | 15.616 (10.1349 to 22.5684) | -0.3197 (-0.5122 to -0.1272) | -1.38 (-1.64 to -1.12) |
| Western Europe | 3975.0459 (2653.5021 to 5920.2542) | 4919.9921 (3074.2577 to 7362.2342) | 0.2377 (-0.1405 to 0.6159) | 34.6248 (23.1135 to 51.5687) | 30.9513 (19.34 to 46.3153) | -0.1061 (-0.3793 to 0.1671) | -0.08 (-0.2-0.04) |
| Western Sub-Saharan Africa | 213.0213 (141.5271 to 305.6497) | 546.4606 (364.5576 to 782.7805) | 1.5653 (0.8546 to 2.276) | 9.0083 (5.9849 to 12.9253) | 7.6441 (5.0996 to 10.9498) | -0.1514 (-0.3865 to 0.0837) | -0.46 (-0.52 to -0.41) |
